# Chinmo prevents *transformer* alternative splicing to maintain male sex identity

**DOI:** 10.1371/journal.pgen.1007203

**Published:** 2018-02-01

**Authors:** Lydia Grmai, Bruno Hudry, Irene Miguel-Aliaga, Erika A. Bach

**Affiliations:** 1 Department of Biochemistry & Molecular Pharmacology, New York University School of Medicine, New York, New York, United States of America; 2 MRC Clinical Sciences Centre, Imperial College London, London, United Kingdom; 3 Kimmel Stem Cell Center, New York University School of Medicine, New York, New York, United States of America; University of California Davis, UNITED STATES

## Abstract

Reproduction in sexually dimorphic animals relies on successful gamete production, executed by the germline and aided by somatic support cells. Somatic sex identity in *Drosophila* is instructed by sex-specific isoforms of the DMRT1 ortholog Doublesex (Dsx). Female-specific expression of Sex-lethal (Sxl) causes alternative splicing of *transformer* (*tra*) to the female isoform *tra*^*F*^. In turn, Tra^F^ alternatively splices *dsx* to the female isoform *dsx*^*F*^. Loss of the transcriptional repressor Chinmo in male somatic stem cells (CySCs) of the testis causes them to “feminize”, resembling female somatic stem cells in the ovary. This somatic sex transformation causes a collapse of germline differentiation and male infertility. We demonstrate this feminization occurs by transcriptional and post-transcriptional regulation of *tra*^*F*^. We find that *chinmo*-deficient CySCs upregulate *tra* mRNA as well as transcripts encoding *tra*-splice factors Virilizer (Vir) and Female lethal (2)d (Fl(2)d). *tra*^*F*^ splicing in *chinmo*-deficient CySCs leads to the production of Dsx^F^ at the expense of the male isoform Dsx^M^, and both Tra^F^ and Dsx^F^ are required for CySC sex transformation. Surprisingly, CySC feminization upon loss of *chinmo* does not require Sxl but does require Vir and Fl(2)d. Consistent with this, we show that both Vir and Fl(2)d are required for *tra* alternative splicing in the female somatic gonad. Our work reveals the need for transcriptional regulation of *tra* in adult male stem cells and highlights a previously unobserved Sxl-independent mechanism of *tra*^*F*^ production *in vivo*. In sum, transcriptional control of the sex determination hierarchy by Chinmo is critical for sex maintenance in sexually dimorphic tissues and is vital in the preservation of fertility.

## Author summary

Sexually dimorphic adult tissues, like ovaries and testes, require continuous sex-specific instruction for proper function. Establishment of female somatic sex identity in *Drosophila* is controlled by an alternative splicing cascade wherein Sex-lethal (Sxl) produces the female-specific protein Transformer^F^ (Tra^F^). By contrast, males lack Sxl and undergo default splicing, preventing Tra^F^ production. Since Tra^F^ expression in males causes sex transformation and impairs tissue function, males must have evolved robust protection against feminization. Here, we investigate the role of a single factor, Chinmo, in protecting male sex identity in the testis: loss of Chinmo in male somatic stem cells causes them to acquire female identity. We demonstrate that this feminization occurs through the induction of Tra^F^ and its downstream targets. Surprisingly, Sxl is not induced in these sex transformed cells. Instead, two other alternative splice factors, Virilizer and Female lethal (2)d, are enriched in *chinmo*-mutant somatic cells and are required for their feminization. Our work demonstrates that transcriptional repression of female-biased alternative splice factors prevents sex transformation in the somatic gonad and that *tra*^*F*^ production can occur independently of Sxl. Given the importance of sex maintenance in tissue homeostasis, such protective mechanisms may exist in other tissues.

## Introduction

Sexual dimorphism, or the differences between male and female individuals in a species, is observed in many organisms, including insects, reptiles, and mammals. Sex-specific tissue development is essential for proper gonadogenesis, and sexual dimorphism has also been observed in other tissues such as brain, adipose tissue, and intestine [1–4]. While extensive literature has dissected the mechanism of sex determination in early development, recent studies have demonstrated that maintenance of sex identity is also essential for adult tissue homeostasis [5–7]. It is therefore critical to determine the signals that both specify and maintain sex identity.

Differential gene expression via alternative splicing establishes the sex-specific differences observed in the fruit fly *Drosophila melanogaster*. In flies, the sex of an organism is determined by its number of X chromosomes [8–10]. In XX flies, a positive autoregulatory mechanism activates and maintains expression of the RNA-recognition motif (RRM) containing protein Sex-lethal (Sxl) [11]. In female somatic cells, Sxl binds directly to a polyuridine (poly(U)) tract upstream of exon 2 in *transformer* (*tra*) pre-mRNA [12, 13]. This results in the skipping of exon 2, which contains an early stop codon, and synthesis of full-length Tra (Tra^F^) in females. In XY flies, which lack Sxl, *tra* mRNA incorporates exon 2, resulting in premature translational termination and a presumptive small peptide with no known function [13]. Several other factors have been shown to act in concert with Sxl in sex-specific alternative splicing, such as Virilizer (Vir), Female lethal (2)d (Fl(2)d), and Spenito (Nito). All three proteins have an RRM and are required for sex-specific and non-sex-specific functions in *Drosophila* [14–19].

One of the best characterized targets of the RNA-binding protein Tra^F^ is *doublesex* (*dsx*), which can yield one of two functional isoforms [20]. In XX flies, Tra^F^ is required for the alternative splicing of *dsx* and *fruitless* (*fru*) pre-mRNAs, generating female-specific Dsx^F^ and preventing Fru synthesis [21, 22]. In XY flies, which lack Tra^F^, *dsx* and *fru* pre-mRNA undergo default splicing and generate male-specific Dsx^M^ and Fru^M^. The Dsx^F^ and Dsx^M^ transcription factors regulate the majority of known sex-specific differences in gene expression and external appearance in *Drosophila*, often by direct transcriptional regulation of critical sex-specific genes [20, 23, 24]. Dsx^F^ and Dsx^M^ have identical DNA binding sites and bind regulatory sites in many common target genes, and it is generally believed that Dsx isoform association with sex-specific co-factors determines whether the target gene is activated or repressed [20, 25–28].

Loss of sex identity in sexually dimorphic tissues has profound effects on organ development and function [1–4, 29–31]. In the gonad, sex identity is specified autonomously in both the germline and the soma; somatic gonadal cells additionally send essential non-autonomous cues to instruct germline sex identity [29, 31–34]. Proper gonadogenesis is impeded when the sex identity of the germline does not match that of the soma, and such a mismatch frequently causes sterility [31, 32]. Despite the importance of maintaining sex identity for tissue development and homeostasis, regulation of canonical sex determinants at the transcriptional level has remained relatively unexplored.

**Fig 1:**
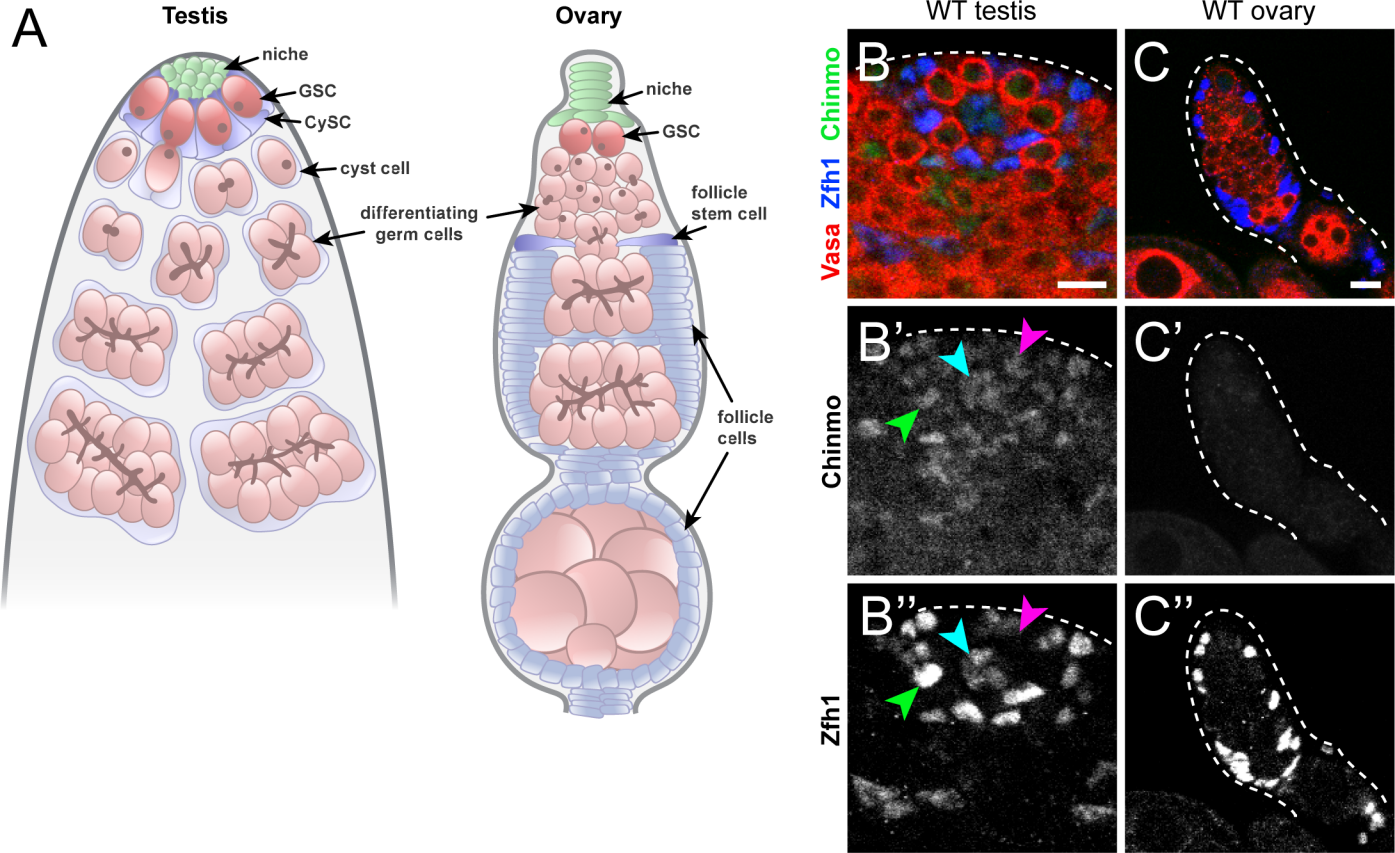
Chinmo is expressed dimorphically in *Drosophila* gonads. (A) Schematic of the adult *Drosophila* testis (left) and ovary (right). In the testis, the niche (green) supports two populations of stem cells, germline stem cells (GSCs, dark pink) and somatic cyst stem cells (CySCs, dark blue). The GSC divides to produce differentiating daughter cells (light pink) that undergo four transit-amplifying divisions. The CySC divides to produce cyst daughter cells (light blue) that exit the cell cycle and ensheath the differentiating GSC daughter. Cyst cells continue to ensheath the associated spermatogonial cyst during transit-amplifying divisions. In the ovary, the niche (green) supports GSCs (dark pink), which divide to give rise to differentiating daughters (light pink) that undergo 4 mitotic divisions. The developing germline cyst is ensheathed by an epithelial layer of follicle cells (light blue). Follicle cells are proliferative descendants of follicle stem cells (FSCs, dark blue) located in the anterior part of the ovary. (B) Chinmo (green) is present in CySCs (B’, green arrowhead), GSCs (B’, magenta arrowhead), and niche cells (B’, cyan arrowhead) in a wild type testis. (C) Chinmo is not expressed in follicle cells of a wild type ovary. Vasa (red) marks the germline and Zfh1 (blue) marks somatic cells in the testis and ovary. Scale bars = 10 μm.

In *Drosophila* gonads, germline stem cells (GSCs) divide to produce daughters that ultimately differentiate into sperm and oocytes, respectively. Proper gametogenesis proceeds through the ensheathment of GSC daughters by somatic support cells that exhibit sex-specific differences. In the testis, a niche of quiescent somatic cells termed the hub supports GSCs and somatic cyst stem cells (CySCs), which produce somatic support cells (Fig 1A, left). GSCs divide with oriented mitosis, and daughter cells that are displaced from the niche differentiate through 4 rounds of transit-amplifying mitotic divisions. CySCs are the only mitotically active somatic cells in wild type testes, and they divide to produce post-mitotic cyst cells. Two cyst cells ensheath a single GSC daughter and remain associated with the germ cell cluster throughout its transit-amplifying divisions. During somatic differentiation, cyst cells grow dramatically to accommodate the enlarging spermatogonia [35–38].

In the ovary, GSCs also divide to produce differentiating daughter cells that undergo 4 mitotic divisions to give rise to 16-cell interconnected germ cysts (Fig 1A, right). The developing germ cyst is surrounded by a layer of somatic follicle cells, which are produced by follicle stem cells (FSCs). CySCs and FSCs require similar self-renewal signals, and both male and female somatic gonadal cells exhibit similar cellular behaviors [39–50]. However, their differentiating offspring exhibit distinct behaviors and markers: cyst cells are quiescent as they differentiate, while follicle cells continue to cycle. Additionally, follicle cells form an epithelium to ensheath the germline, while cyst cells grow in volume and express tight junction proteins to encapsulate spermatogonia [35, 37, 38, 51–53].

Sex-specific anatomical differences are achieved by differential expression of transcription factors [2]. In particular, the transcription factor Chinmo is expressed in male but not female somatic gonadal cells [29, 54, 55]. Chinmo contains a Broad, Tramtrack, and Bric-à-brac/Poxvirus and Zinc finger (BTB/POZ) domain and two C_2_H_2_-Zinc fingers (ZFs). Many BTB-ZF proteins in *Drosophila* and mammals have characterized roles as transcriptional repressors [56–58]. However, while clonal loss of *chinmo* from imaginal tissue leads to ectopic gene expression in a cell-autonomous manner [54], no direct targets of Chinmo have been identified. Congruent with its dimorphic expression in the somatic gonad, *chinmo* has no apparent requirement in follicle cells but is essential for CySC niche occupancy [54, 55]. Chinmo is also required for the maintenance of male sex identity in CySCs, as loss of *chinmo* from all CySCs causes them to lose male sex identity, express markers of ovarian follicle cells and adopt an epithelial-like organization [29]. These data have led to a model in which single CySC clones lacking *chinmo* are outcompeted by wild type CySC neighbors, but *chinmo* depletion in all CySCs removes this competitive environment and leads to sex transformation [29, 54]. We have also observed that *chinmo-*mutant CySC clones that lack the JAK/STAT and EGFR pathway inhibitor *Socs36E* can form aggregates, suggesting that CySCs lacking *chinmo* can feminize so long as they are given a chance to proliferate [59]. This sex transformation was reportedly due in part to a transcriptional loss at the *dsx* locus, leading to a loss of Dsx^M^; however, sustained expression of UAS-*dsx*^*M*^ could not prevent the acquisition of female sex identity in *chinmo*-mutant CySCs, indicating that the molecular mechanism by which these cells feminize is still unclear [29].

Our work supports an alternate model whereby male sex identity is maintained not by preventing transcriptional loss of *dsx*^*M*^, but by preventing alternative splicing of *dsx* pre-mRNA into *dsx*^*F*^. Since Tra^F^ is responsible for *dsx* alternative splicing in canonical sex determination, we investigated a possible role for Tra^F^ in CySC feminization upon *chinmo* loss. Here, we report that Chinmo maintains male sexual identity by preventing the expression of the female sex determinant *tra*^*F*^ through a two-step mechanism. We first show that Chinmo represses both expression and alternative splicing of *tra* pre-mRNA. Next, we demonstrate that feminization of *chinmo*-mutant CySCs does not require Sxl. We instead find that RNA binding proteins Vir and Fl(2)d, which are necessary to alternatively splice *tra*^*F*^ in the adult ovary, are important for the feminization of *chinmo*-mutant CySCs. Thus, we uncover a novel mode of sex maintenance involving previously unreported regulation of *tra* transcription and a Sxl-independent mechanism of *tra*^*F*^ splicing in the somatic gonad.

## Results

### Chinmo is expressed dimorphically in the somatic gonad and is required for male identity in CySCs

**Fig 2:**
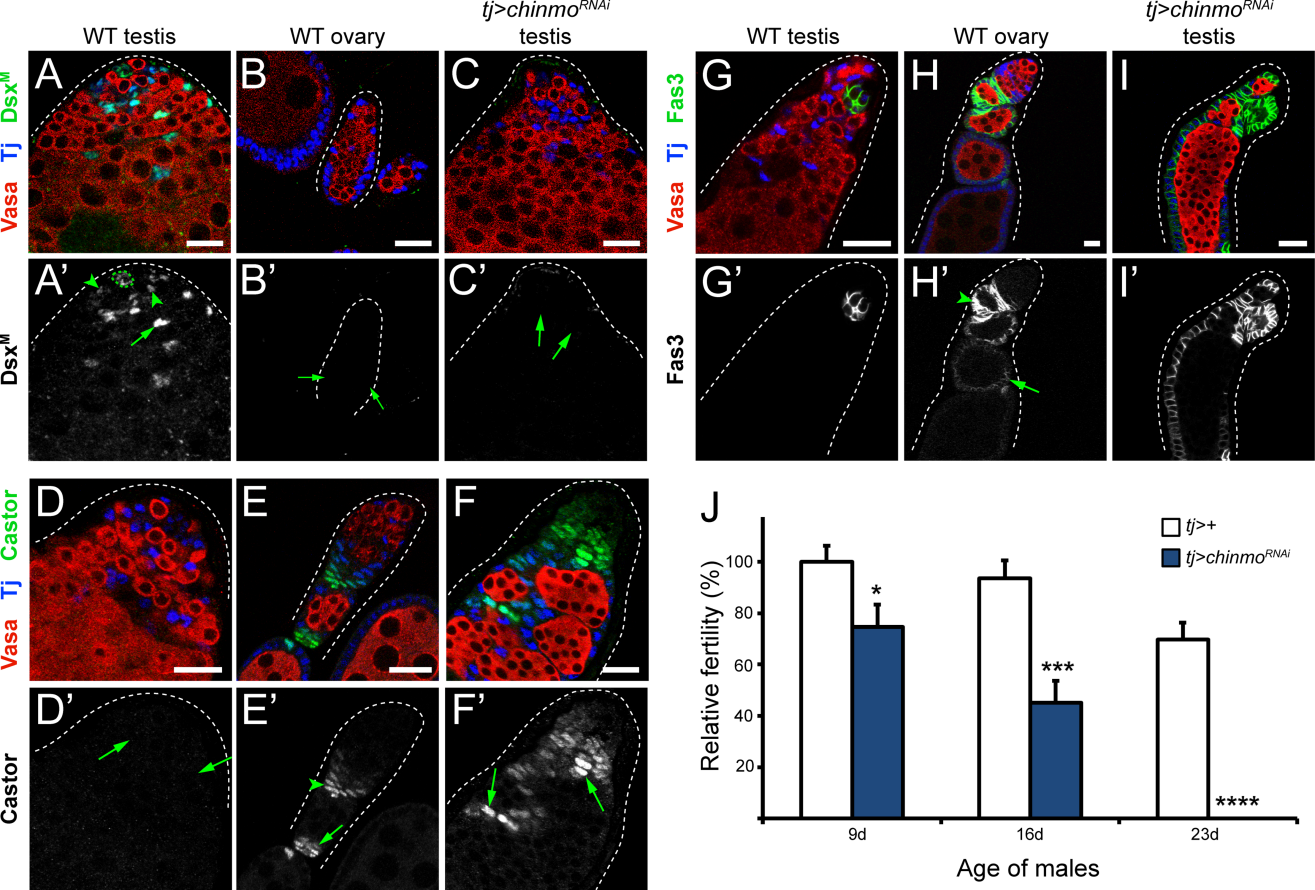
Chinmo is required in CySCs for male somatic sex identity and non-autonomously for germline maintenance. (A) In a wild type testis, Dsx^M^ (green) is present in niche cells (A’, outlined with dotted green line), CySCs (A’, arrowheads) and cyst cells (A’, arrow). (B) Dsx^M^ is not expressed in a wild type ovary (B’, arrows indicate early follicle cells). (C) Dsx^M^ is lost in the CySC lineage in a *tj>chinmo^RNAi^* testis (C’, arrows indicate early somatic cells). (D) Castor (Cas, green) is absent from a wild type testis (D’, arrows indicate early somatic cells). (E) In a wild type ovary, Cas (green) is expressed in early follicle cells (E’, arrowhead) and stalk cells (E’, arrow). (F) Cas (green) is ectopically expressed in feminizing somatic cells in the testis (F’, arrows) upon loss of Chinmo. (G) Fasciclin-3 (Fas3, green) is normally restricted to niche cells in a wild type testis. (H) In a wild type ovary, Fas3 (green) is high in early follicle cells (H’, arrowhead) and is lower in mature follicle cells (H’, arrow). (I) Fas3 (green) is ectopically expressed upon loss of Chinmo in CySC lineage. (J) Relative fertility of *tj>chinmo*^*RNAi*^ males (blue bars) is decreased at 9, 16 or 23 days (d) post eclosion compared with control *tj>*+ males (white bars). *tj>chinmo*^*RNAi*^ males are completely sterile by 23 days post eclosion (* denotes p<0.05; *** denotes p<0.001; **** denotes p<0.0001 as determined by single-factor ANOVA). Error bars represent SEM. In A-I, Vasa (red) marks the germline and Tj (blue) marks cyst cells. Scale bars = 20 μm. Time point in A-I is 7 days post-eclosion.

We found dimorphic expression of Chinmo in *Drosophila* gonads. While Chinmo protein was expressed in all cell types of the adult testis stem cell niche (Fig 1B and 1B’, arrowheads; S1I Fig), it was not detectable in somatic cells of the adult ovary (Fig 1C; S1J Fig). We next confirmed that loss of Chinmo expression in CySCs leads to the acquisition of female identity. When *chinmo* was depleted in the CySC lineage by RNAi using the somatic driver *tj-gal4* (*tj>chinmo*^*RNAi*^; S1K Fig), expression of the male sex determinant Dsx^M^ was lost (Fig 2A–2C), and the follicle cell marker Castor (Cas), normally absent from the testis, was ectopically expressed (Fig 2D–2F). In wild type testes, Fasciclin 3 (Fas3) was expressed in niche cells but not in CySCs (Fig 2G). However, in *tj>chinmo*^*RNAi*^ testes, we observed Fas3-expressing somatic aggregates resembling epithelial follicle cells that eventually organized at the periphery (Fig 2H and 2I). A marker of late-stage follicle cell maturation, Slow border cells (Slbo), was absent from wild type CySCs (S1A and S1B Fig) but was ectopically expressed in *tj>chinmo*^*RNAi*^ testes (S1C Fig). Finally, transcripts of the Dsx^F^ target *Yp1* were upregulated in *chinmo*-deficient testes (S1D Fig; [20]. This sex transformation phenotype is due to loss of *chinmo* in the CySC lineage and not the niche, as depletion of *chinmo* specifically in niche cells produced no overt phenotype (S1E and S1F Fig; [29]).

Because CySCs serve a critical role in maintaining GSCs, as well as producing somatic support cells, the stem cell niche in *tj>chinmo*^*RNAi*^ testes frequently becomes agametic even at relatively early time points after depletion (S1G and S1H Fig; [29, 60]). Based upon these observations, we hypothesized that *tj>chinmo*^*RNAi*^ males would become sterile. To test this, we mated successively *tj>chinmo*^*RNAi*^ males to *Oregon*^*R*^ virgin females and scored the number of progeny. Upon each of two mating rounds, *tj>chinmo*^*RNAi*^ males exhibited a significant reduction in fertility (25% and 55% compared to control males, p<0.05 and p<0.001, respectively) (Fig 2J). By the third successive mating, *tj>chinmo*^*RNAi*^ males were completely sterile whereas control males were not (p<0.0001). Taken together, our results align with previous work showing that Chinmo is required in adult CySCs to preserve male sex identity [29]. Additionally, we demonstrate that CySC male identity is essential for fertility.

### CySC feminization upon loss of *chinmo* is dependent on the female sex determinant *dsx*^*F*^

**Fig 3:**
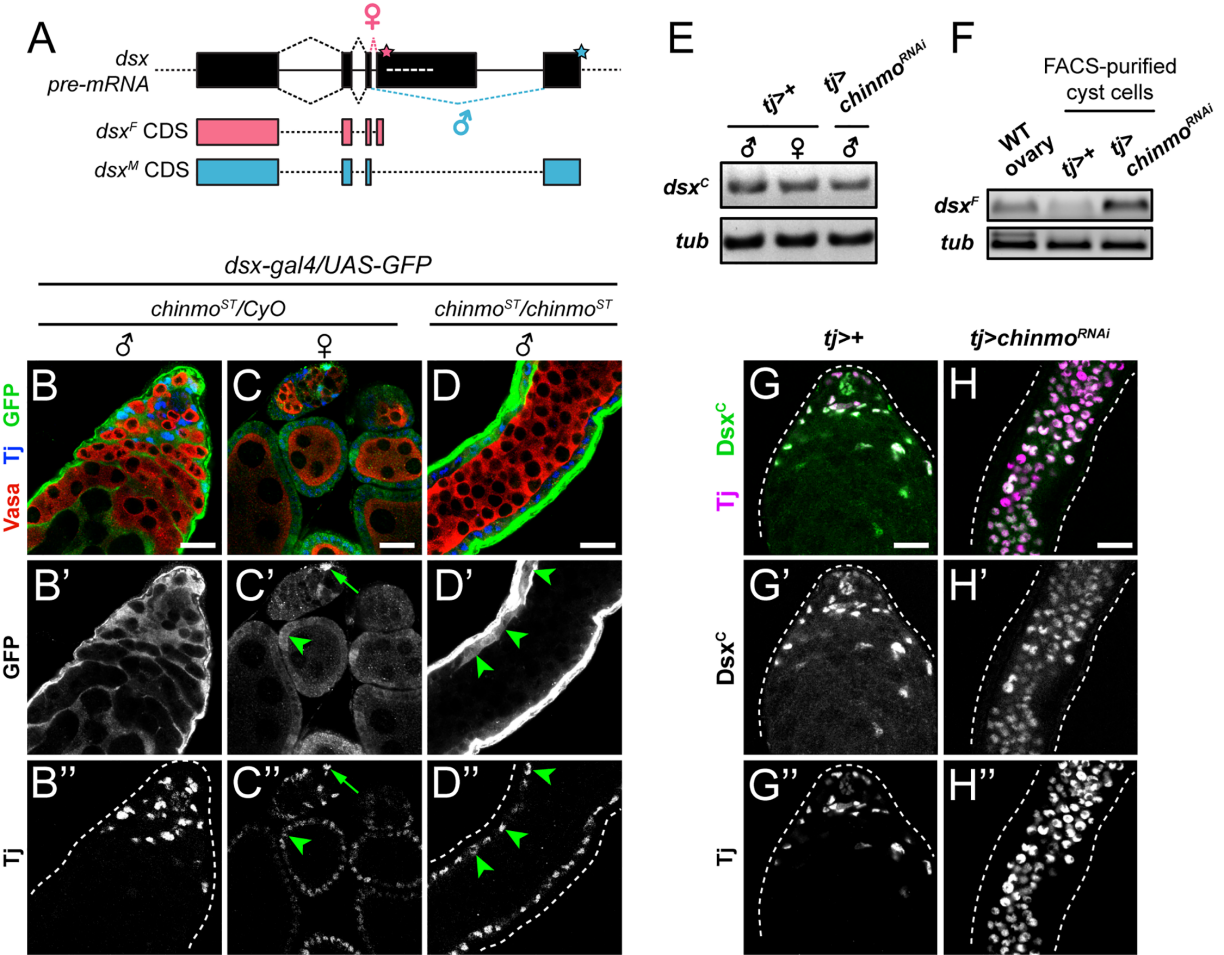
Dsx^F^ protein is synthesized in *chinmo*-mutant CySCs. (A) Schematic of *dsx* pre-mRNA splicing. *dsx*^*F*^ and *dsx*^*M*^ share the first three coding exons and differ at their C-termini. Exon 4 contains a non-canonical splice acceptor, and thus under default splicing conditions, exon 3 is adjoined with exon 5 to yield the male-specific *dsx*^*M*^ isoform (blue). In XX somatic cells, Tra^F^ is expressed and binds tandem Tra^F^-binding sites (white dashed line) in exon 4. The Tra^F^ complex then recruits the spliceosome, leading to synthesis of the female-specific *dsx*^*F*^ isoform (pink). Pink and blue stars indicate female and male stop codons, respectively. (B) A knock-in transcriptional reporter for *dsx* (*dsx-gal4*) activates UAS-GFP expression (green) in the CySC lineage of a wild type testis. (C) *dsx-gal4* activates GFP expression in both escort cells (C’, arrow) and follicle cells (C’, arrowheads) of wild type ovary. (D) *dsx-gal4* activates GFP expression (D’, arrowheads) in the somatic lineage of a *chinmo*^*ST*^*/chinmo*^*ST*^ testis. Note that the feminized soma in this testis is organized at the periphery, adjacent to the muscle sheath. (E) Semi-quantitative RT-PCR on homogenized wild type testes (left lane), wild type ovaries (middle lane), and *tj>chinmo*^*RNAi*^ testes (right lane). As determined by primers that recognize both *dsx* mRNA isoforms (*dsx*^*COMMON*^, or *dsx*^*C*^), *dsx* transcripts are present in both wild type testes and wild type ovaries (left and middle lanes). *dsx* mRNA is also expressed in *tj>chinmo*^*RNAi*^ testes (right lane). *α-tub* (*tub*) was used as a loading control. Flies were aged 9–20 days prior to dissection. (F) Semi-quantitative RT-PCR on RNA extracts from FACS-purified cyst cells from control *tj>+* or *tj>chinmo*^*RNAi*^ testes. Whole adult ovaries from *yw* females (labeled “WT ovary”) were homogenized as a positive control for *dsx*^*F*^ mRNA detection. *dsx*^*F*^ is detected in wild type ovaries (left lane) and in *tj>chinmo*^*RNAi*^ cyst cells (right lane) but not in *tj>+* cyst cells (middle lane). *β-tubulin* (*tub*) was used as a loading control. (G,H) Dsx (green), as detected by the Dsx^C^ antibody that recognizes both Dsx^F^ and Dsx^M^, is present in somatic cells of a wild type testis (G) and a *tj>chinmo*^*RNAi*^ testis (H). Tj (magenta) marks cyst cells. In B-D, Vasa (red) marks germ cells and Tj (blue) marks cyst cells. Scale bars = 20 μm.

We next sought to determine the mechanism by which CySCs undergo feminization upon loss of *chinmo*. According to a previous report, *dsx*^*M*^ mis-expression in *chinmo*-deficient CySCs (*c587>chinmo*^*RNAi*^*; >dsx*^*M*^) delays feminization [29], suggesting that *dsx*^*M*^ transcription was reduced in *chinmo*-mutant CySCs. However, at the time point when all *c587>chinmo*^*RNAi*^ testes contained Fas3-positive aggregates, nearly all *c587>chinmo*^*RNAi*^*; >dsx*^*M*^ testes were also feminized [29], indicating a delay but not an abrogation of the phenotype. Additionally, depletion of all *dsx* transcripts using an RNAi transgene (*dsx*^*KK111266*^) targeting the common region of *dsx*^*M*^ and *dsx*^*F*^ did not recapitulate the defect seen upon loss of *chinmo* [29]. These data indicate that the loss of Dsx^M^ alone cannot fully account for the phenotype of *chinmo*-mutant CySCs. We reasoned that the loss of Dsx^M^ protein observed in *chinmo*-mutant CySCs could result from alternative splicing of the *dsx* pre-mRNA into *dsx*^*F*^ rather than from a transcriptional decrease at the *dsx* locus (Fig 3A). If this were true, we would expect to find in *chinmo*-deficient CySCs: 1) active transcription of the *dsx* locus; 2) expression of alternatively-spliced *dsx*^*F*^ transcripts; and 3) expression of Dsx^F^ protein. To assess *dsx* transcription levels, we surveyed 4 independently-generated *dsx* transcriptional reporters: two Gal4 knock-in reporters in the *dsx* locus (*dsx-gal4*; [2] and *dsx-gal4*^*Δ2*^; [61]), one MiMIC allele at the *dsx* locus (*dsx*^*MI03050-GFSTF*.*1*^; [62]) and one Janelia transgene containing a 2.5 kb *dsx* regulatory element (*GMR40A05-gal4*; [63]. We selected *dsx-gal4* for further use because it was the only line that was robustly expressed in both adult testes and adult ovaries and therefore accurately reflected *dsx* transcription (Fig 3B and 3C). By contrast, the other 3 lines displayed male-biased or very low expression in gonads (S2A–S2F Fig). We then assessed *dsx-gal4* activity as a proxy for transcription of the *dsx* locus upon *chinmo* depletion. We used a genetic approach to remove *chinmo* from the CySC lineage by analyzing testes homozygous for the *chinmo*^*ST*^ allele [29] in the *dsx-gal4* background. While *chinmo*^*ST*^*/CyO* testes express normal levels of Chinmo, *chinmo*^*ST*^*/chinmo*^*ST*^ males lack Chinmo in the CySC lineage [29]. As expected, GFP was expressed in somatic cells in control *chinmo*^*ST*^*/CyO; dsx-gal4/UAS-GFP* testes and ovaries (Fig 3B and 3C). Importantly, GFP was also expressed in *chinmo*^*ST*^*/chinmo*^*ST*^; *dsx-gal4/UAS-GFP* mutant testes (Fig 3D), demonstrating that *dsx* is still transcribed in *chinmo*-deficient cyst cells. We also visualized *dsx* transcript abundance in *tj>chinmo*^*RNAi*^ testes using semi-quantitative RT-PCR. Primers that recognize both *dsx* mRNA isoforms (*dsx*^*COMMON*^, or *dsx*^*C*^) reveal that *dsx* is still present in *tj>chinmo*^*RNAi*^ testes (Fig 3E). We confirmed that *dsx*^*F*^ is produced specifically by *chinmo*-deficient somatic cells by performing RT-PCR on FACS-sorted CySCs and early cyst cells. As expected, a *dsx*^*F*^-specific band was observed in RNA extracts from wild type ovaries (Fig 3F, left lane). We also observed *dsx*^*F*^ in FACS-purified *chinmo*-deficient cyst cells (Fig 3F, right lane). As expected, *dsx*^*F*^ was absent from FACS-purified wild type cyst cells (Fig 3F, middle lane).

We next visualized Dsx protein in control *tj>+* and *tj>chinmo*^*RNAi*^ testes using an antibody that detects both isoforms of Dsx (anti-Dsx^C^; [64]). We observed that Dsx protein is still synthesized in somatic cells lacking *chinmo* (Fig 3G and 3H); because Dsx^M^ is lost from *tj>chinmo*^*RNAi*^ testes (Fig 2C), we conclude that the Dsx protein present in *chinmo*-deficient somatic cells is Dsx^F^. We confirmed that anti-Dsx^C^ detects Dsx^F^ by staining *tj>dsx*^*F*^ ovaries (S3 Fig). These results suggest that Dsx^M^ loss in *chinmo*-deficient CySCs is not due to transcriptional loss of *dsx*^*M*^, but rather alternative splicing that generates the female isoform Dsx^F^.

**Fig 4:**
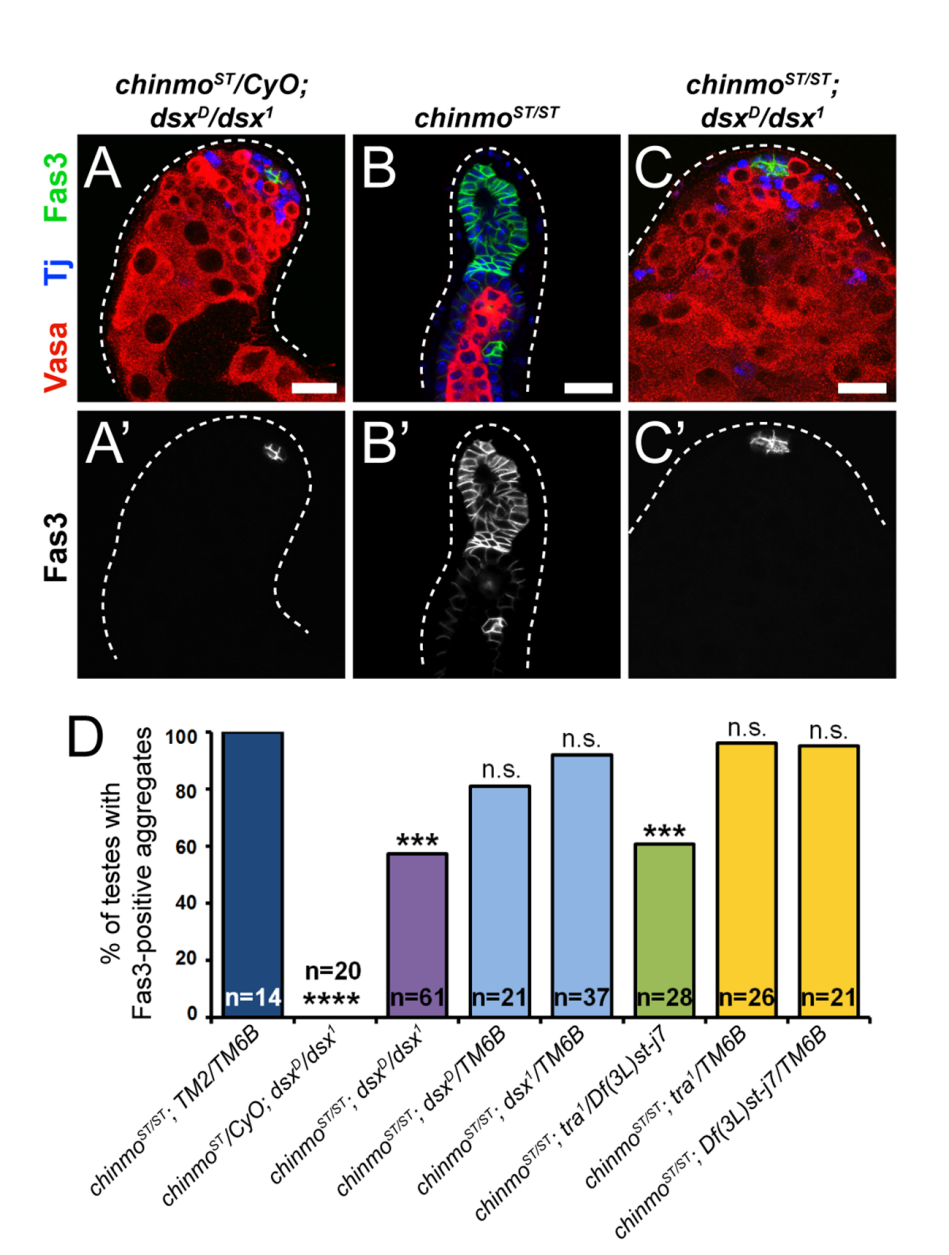
Dsx^F^ is required for feminization in *chinmo*-mutant CySCs. (A-C) Representative confocal images of testes from *chinmo*^*ST*^*/chinmo*^*ST*^; *dsx*^*D*^*/dsx*^*1*^ flies. In a testis from a *chinmo*^*ST/*^*CyO*; *dsx*^*D*^*/dsx*^*1*^ sibling male, only niche cells express Fas3 (A’). By contrast, in a *chinmo*^*ST*^*/chinmo*^*ST*^ testis most of the somatic lineage is positive for Fas3 (B’). In a testis from a “rescued” *chinmo*^*ST*^*/chinmo*^*ST*^; *dsx*^*D*^*/dsx*^*1*^ male, Fas3 is again restricted to niche cells (C’). Results are quantified in Fig 4D and S1 Table. Vasa (red) marks germ cells and Tj (blue) marks cyst cells. Scale bars = 20 μm.(D) Quantification of Fas3-positive aggregates as a readout of feminization. 100% of *chinmo*^*ST*^*/chinmo*^*ST*^; *TM2/TM6B* testes contain Fas3-positive aggregates (dark blue bar) at 7 days post-eclosion. By contrast, none of the testes from *chinmo*^*ST*^*/CyO*; *dsx*^*D*^*/dsx*^*1*^ sibling males contain aggregates (second bar). There is a significant reduction in the percentage of testes from *chinmo*^*ST*^*/chinmo*^*ST*^; *dsx*^*D*^*/dsx*^*1*^ (purple bar) or *chinmo*^*ST*^*/chinmo*^*ST*^; *tra*^*1*^*/Df(3L)st-j7* (green bar) containing Fas3-positive aggregates, indicating a partial rescue of the feminization. However, testes from the various sibling controls are still feminized (light blue and yellow bars). Sample sizes are indicated within bars. *** denotes p<0.001 and **** denotes p<0.0001 as determined by two-tailed Student’s t-test (for qRT-PCR, compared with *tj>+* controls) or Fisher’s Exact Test (for quantifications, compared with *chinmo*^*ST*^*/chinmo*^*ST*^). n.s. means not significant. See S1 Table for percentage values.

We next tested whether Dsx^F^ production is causal to feminization of CySCs lacking *chinmo*. We took a genetic approach and blocked *dsx*^*F*^ splicing by using mutant alleles *dsx*^*D*^*/dsx*^*1*^. *dsx*^*D*^ cannot be alternatively spliced into *dsx*^*F*^ but produces normal levels of *dsx*^*M*^, and *dsx*^*1*^ is a null allele. *dsx*^*D*^*/dsx*^*1*^ flies only produce Dsx^M^. XX *dsx*^*D*^*/dsx*^*1*^ animals develop male abdominal pigmentation, genitalia, and sex combs due to a masculinized soma (S4A–S4D Fig; [65]). We introduced *dsx*^*D*^*/dsx*^*1*^ into males homozygous for the *chinmo*^*ST*^ allele [29]. As expected, control *chinmo*^*ST*^*/CyO; dsx*^*D*^*/dsx*^*1*^ sibling testes appeared normal (Fig 4A; Fig 4D, second bar; S1 Table). By contrast, 100% of *chinmo*^*ST*^*/chinmo*^*ST*^*; TM2/TM6B* testes at 7 days post-eclosion contained Fas3-positive somatic aggregates outside the hub (Fig 4B; Fig 4D, first bar; S1 Table). Strikingly, only 57% of *chinmo*^*ST*^*/chinmo*^*ST*^; *dsx*^*D*^*/dsx*^*1*^ testes contained Fas3-positive aggregates (Fig 4C; Fig 4D, purple bar; S1 Table), a significant reduction compared to *chinmo*^*ST*^*/chinmo*^*ST*^ flies (p<0.001). Taken together, our results reveal that (1) acquisition of female identity in *chinmo*-mutant CySCs occurs by *dsx* alternative splicing that generates Dsx^F^ and (2) *dsx*^*F*^ production is required in part for CySC feminization upon loss of *chinmo*.

### Tra^F^ is required for feminization in CySCs lacking *chinmo*

**Fig 5:**
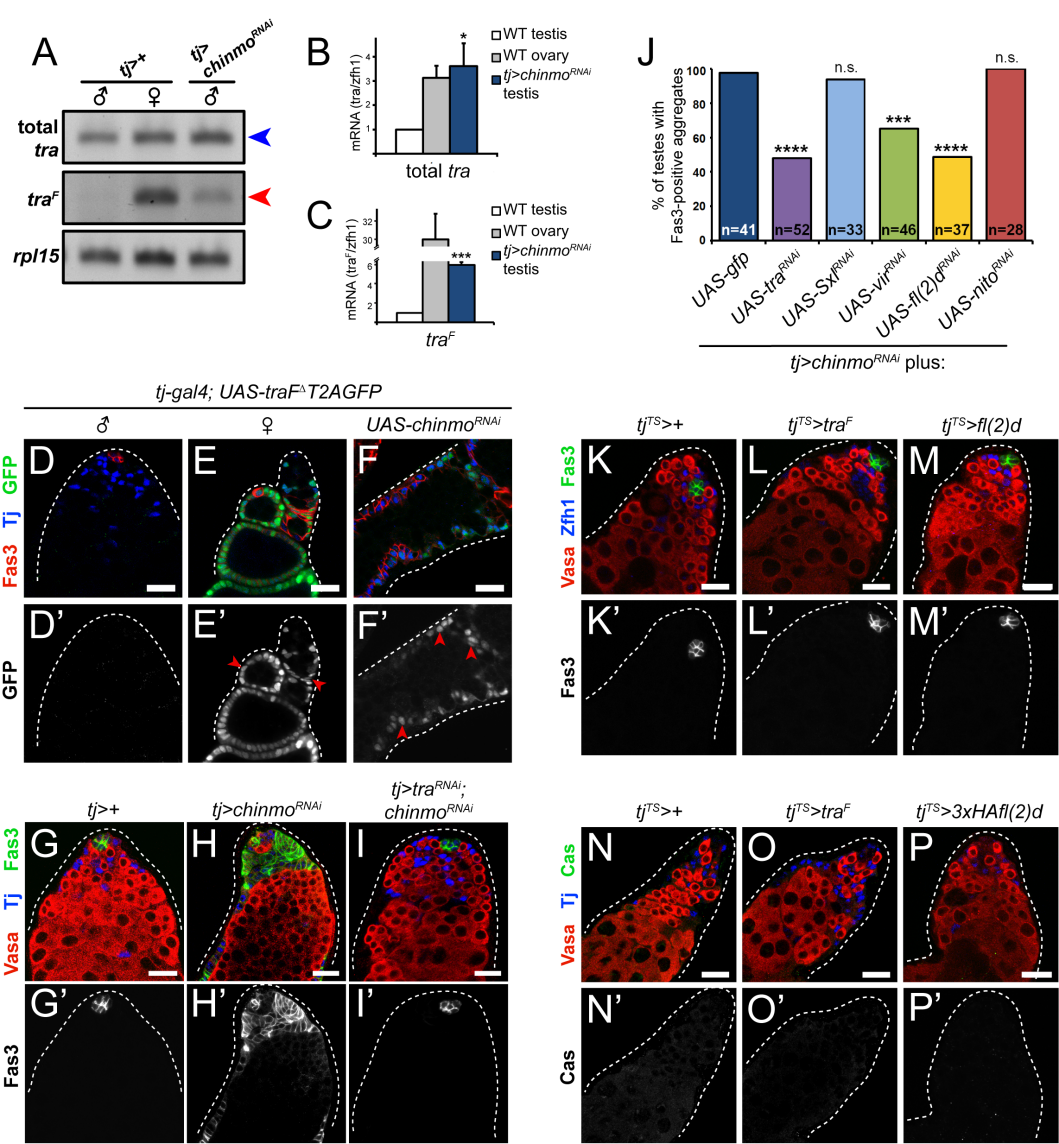
The female sex determinant Tra^F^ is required for feminization of *chinmo*-mutant CySCs. (A) Semi-quantitative RT-PCR of total *tra* and *tra*^*F*^ mRNA in control *tj>+* testes (first lane), control *tj>+* ovaries (second lane) and *tj>chinmo*^*RNAi*^ testes (third lane). All three samples contain a band for total *tra* (first row, blue arrowhead). However, a *tra*^*F*^ band is detected in ovaries, as expected, and in *tj>chinmo*^*RNAi*^ testes (second row, red arrowhead) but not in control *tj>+* testes. *rpl15* was used as a loading control (third row). Flies were aged 9–20 days prior to dissection. (B-C) qRT-PCR of total *tra* (B) or *tra*^*F*^ (C) in control *tj>+* testes (white bars), control *tj>+* ovaries (gray bars) and *tj>chinmo*^*RNAi*^ testes (blue bars). There is significantly more total *tra* mRNA (B) and *tra*^*F*^ (C) in *tj>chinmo*^*RNAi*^ testes compared to control *tj>+* testes. Values represent the average of three biological replicates. * denotes p<0.05; *** denotes p<0.001 as determined by two-tailed Student’s t-test (compared with *tj>+* testes). Error bars represent SEM. Flies were aged 9–20 days prior to dissection. (D-F) GFP caused by alternative splicing of *traF*^*Δ*^*T2AGFP* pre-mRNA is not observed in control *tj>traF*^*Δ*^*T2AGFP* testes (D’). By contrast, GFP indicative of *tra*^*F*^ splicing is robustly observed in follicle cells of a control *tj>traF*^*Δ*^*T2AGFP* ovary (E’, arrowheads) and in somatic cells of a *tj>traF*^*Δ*^*T2AGFP; chinmo*^*RNAi*^ testis (F’, arrowheads). Tj (blue) marks somatic cells. Fas3 (red) marks hub cells in wild type testes, follicle cells in wild type ovaries and feminized cyst cells in *tj>chinmo*^*RNAi*^ testes. (G-I) In a control *tj>+* testis, only niche cells express Fas3 (G’). By contrast, in a *tj>chinmo*^*RNAi*^ testis, most of the somatic lineage is positive for Fas3 (H’). In a “rescued” *tj>tra*^*RNAi*^*; chinmo*^*RNAi*^ testis, Fas3 is again restricted to niche cells (I’). Vasa (red) marks germ cells, Tj (blue) marks cyst cells, and Fas3 (green) marks niche cells and feminizing somatic cells. Results are quantified in Fig 4J and S1 Table. (J) Quantification of CySC feminization in *tj>GFP; chinmo*^*RNAi*^ testes and various genotypes. 100% of *tj>GFP; chinmo*^*RNAi*^ testes contain Fas3-positive aggregates (dark blue bar). There is a significant reduction in the percentage of feminized testes when *tra* is concomitantly depleted from *tj>chinmo*^*RNAi*^ testes (purple bar). There is also a significant reduction when *vir* or *fl(2)d* is depleted from *tj>chinmo*^*RNAi*^ testes (green and yellow bars, respectively). However, there is no rescue of male sex identity in *tj>chinmo*^*RNAi*^ testes when *Sxl*^,^ or *nito* is concomitantly depleted (light blue and red bars, respectively). Sample sizes are indicated within bars. *** denotes p<0.001; **** denotes p<0.0001 as determined by Fisher’s Exact Test (for rescue quantifications, compared with *tj>GFP; chinmo*^*RNAi*^). n.s. means not significant. See S1 Table for percentage values. (K-M) Fas3 is not ectopically expressed in CySCs from control (*tj*^*TS*^*>+*) testes (K’), testes with somatic mis-expression of *tra*^*F*^ (*tj*^*TS*^*>tra*^*F*^) (L’), or testes with somatic mis-expression of *fl(2)d* (*tj*^*TS*^*>fl(2)d*) (M’). (N-P) Cas is not expressed in control (*tj*^*TS*^*>+*) (N’), *tj*^*TS*^*>tra*^*F*^ testes (O’), or *tj*^*TS*^*>fl(2)d* testes (P’). In K-P, Vasa (red) marks germ cells, and Zfh1 or Tj (blue) mark cyst cells. Scale bars = 20 μm.

Given that Dsx^F^ is produced in *chinmo*-deficient CySCs, these cells must also express a factor that promotes alternative splicing of *dsx* pre-mRNA. In female somatic cells, this alternative splicing is mediated by Tra^F^ [22]. We hypothesized that *tj>chinmo*^*RNAi*^ testes express ectopic Tra^F^ that produces *dsx*^*F*^. To test this, we performed semi-quantitative (Fig 5A) and quantitative RT-PCR (Fig 5B and 5C) analysis on the *tra* locus in *tj>chinmo*^*RNAi*^ testes. We found that total *tra* mRNA abundance significantly increased (3.6-fold) in *tj>chinmo*^*RNAi*^ testes compared with *tj>+* testes (p<0.05) (Fig 5A, blue arrowhead; Fig 5B, compare blue to white bar). Furthermore, *tra*^*F*^-specific primers revealed a 6.1-fold enrichment of *tra*^*F*^ mRNA in *tj>chinmo*^*RNAi*^ testes compared to *tj>+* controls (p<0.001) (Fig 5A, red arrowhead; Fig 5C, compare blue to white bar). These data demonstrate that the ectopic *tra* in *chinmo*-deficient CySCs is indeed spliced into the female *tra*^*F*^ isoform.

To confirm these results, we monitored *tra* alternative splicing *in vivo* using a transgene that yields GFP expression when *tra* pre-mRNA is alternatively spliced (*UAS-traF*^*Δ*^*T2AGFP*). In this transgene, the third exon of *tra* (which is adjoined with exon 1 in the female isoform) is replaced by the coding sequences for self-cleaving T2A peptide and GFP (S5 Fig). As expected, we detected little to no GFP expression in wild type (male) cyst cells (Fig 5D), while wild type (female) follicle cells expressed high levels (Fig 5E’, arrowheads). Notably, we also observed high levels of GFP in the soma of *tj>chinmo*^*RNAi*^ testes (Fig 5F’, arrowheads), demonstrating that *tra* pre-mRNA is alternatively spliced to *tra*^*F*^ in these feminized somatic cells. We conclude that Chinmo normally represses *tra* transcription and alternative splicing in the male somatic gonad.

To determine if ectopic Chinmo is sufficient to repress *tra* transcription, we mis-expressed it in adult ovarian follicle cells using *tj-gal4*. To evade lethality caused by Chinmo mis-expression [54], we used a temperature-sensitive *gal80* allele *(tj-gal4*, *tub-gal80*^*TS*^ *or tj*^*TS*^*)* and reared flies at the permissive temperature (18°C). Adult F1 females were then shifted to the restrictive temperature (29°C) for 5 days before ovaries were homogenized. We observed a 2.2-fold decrease in total *tra* mRNA abundance (p<0.001) and a 1.8-fold decrease in *tra*^*F*^ abundance (p<0.001) in *tj*^*TS*^*>chinmo* ovaries compared with *tj*^*TS*^*>+* ovaries (S6A Fig). *dsx*^*F*^ was also decreased 5.8-fold (p<0.001) in *tj*^*TS*^*>chinmo* ovaries compared with *tj*^*TS*^*>+* ovaries, presumably as a result of reduced Tra^F^ (S6A Fig). Taken together, our results demonstrate that Chinmo is both necessary and sufficient to prevent somatic expression of the female sex determinants *tra*^*F*^ and *dsx*^*F*^.

These findings suggest that sex transformation in *chinmo*-deficient cyst cells is due to ectopic Tra^F^. To test this, we concomitantly depleted both *tra* and *chinmo* in the somatic lineage of the testis. Whereas 98% of *tj>chinmo*^*RNAi*^ testes contained Fas3-positive aggregates outside of the niche, only 48% of *tj>tra*^*RNAi*^*; chinmo*^*RNAi*^ testes had such aggregates, indicating a significant block in feminization (p<0.0001) (Fig 5I; Fig 5J, purple bar; S1 Table). In these rescued *tj>tra*^*RNAi*^*; chinmo*^*RNAi*^ testes, CySCs no longer expressed Fas3, and the germline appeared normal (Fig 5I). We also performed epistatic experiments with *tra* mutant alleles, similar to the *dsx*^*D*^*/dsx*^*1*^ experiment. XX *tra*^*1*^*/Df(3L)st-j7* animals develop male somatic structures due to loss of Tra^F^ (S4E–S4H Fig; [66]). Whereas 100% of *chinmo*^*ST*^*/chinmo*^*ST*^ testes were feminized as assessed by Fas3-positive aggregates, only 61% of *chinmo*^*ST*^*/chinmo*^*ST*^; *tra*^*1*^*/Df(3L)st-j7* testes were feminized (p<0.001) (Fig 4D, green bar; S1 Table). The phenotype was not sensitive to *tra* dose as *chinmo*^*ST*^*/chinmo*^*ST*^; *tra/+* testes were still feminized (Fig 4D, yellow bars; S1 Table). These results demonstrate that Chinmo prevents both *tra* transcription and alternative splicing in CySCs and that feminization of male somatic cells in the absence of *chinmo* is due to ectopic *tra*^*F*^.

### Ectopic Tra^F^ impairs somatic differentiation but is not sufficient for CySC feminization

Global expression of Tra^F^ in XY flies during development causes female somatic differentiation [67]. To test whether Tra^F^ expression alone is sufficient to cause male-to-female sex transformation in adult CySCs, we over-expressed *tra*^*F*^ cDNA in *tj*-*gal4* expressing cells and used *gal80*^*TS*^ to restrict expression to only adult CySCs (*tj*^*TS*^). While we observed accumulation of somatic aggregates in *tj*^*TS*^*>tra*^*F*^ testes, they did not express Fas3 or Cas, in contrast to those in *tj>chinmo*^*RNAi*^ testes (compare Fig 5K and 5L to Fig 2I for Fas3 and compare Fig 5N and 5O to Fig 2F for Cas). These data suggest that *tra*^*F*^-misexpressing cyst cells have not fully acquired a follicle-like fate. However, we found on average 121.0±8.8 somatic cells expressing Zinc finger homeodomain 1 (Zfh1), which marks CySCs and their earliest differentiating daughters [68], in *tj*^*TS*^*>tra*^*F*^ testes compared with 40.1±1.6 cells in control *tj*^*TS*^*>+* testes (p<0.0001) (S7A, S7B and S7I Fig). Upon somatic *tra*^*F*^ mis-expression, we also observed accumulation of somatic cells expressing Tj, which marks a broader population of CySCs and early cyst cells [69] (S7C and S7D Fig). *tj*^*TS*^*>tra*^*F*^ testes contained 158.7±14.5 Tj-positive cells compared with 80.3±3.9 cells in *tj*^*TS*^*>+* testes (p<0.001) (S7J Fig). We interpret the accumulation of Zfh1-positive, Tj-positive cells in *tj*^*TS*^*>tra*^*F*^ testes as a delay in somatic differentiation. Because cyst cells must exit the cell cycle in order to support the developing male germline, there are no somatic cells located away from the niche in wild type testes that are positive for 5-ethynyl-2’-deoxyuridine (EdU), an S-phase marker. We previously showed that when somatic differentiation is delayed, EdU-positive cyst cells are observed several cell diameters away from the niche [36]. Consistent with our prior results, in control *tj*^*TS*^*>+* testes, only Tj-positive cells near the hub incorporated EdU (S7E Fig, arrowheads; n = 0/20 testes with EdU-positive cyst cells located away from the niche). By contrast, in *tj*^*TS*^*>tra*^*F*^ testes we detected EdU-positive somatic cells located many cell diameters away from the niche, suggesting that these cells had delayed differentiation (S7F Fig, arrows; n = 20/26 testes with EdU-positive cyst cells located away from the niche).

Consistent with a defect in somatic differentiation, cyst cells mis-expressing *tra*^*F*^ were impaired in their ability to support the germline. In *tj*^*TS*^*>tra*^*F*^ testes, early germ cells accumulated (identified by dot- and dumbbell-shaped α-spectrin-positive fusomes) at the expense of more differentiated spermatogonia, as fewer germ cysts with branched fusomes were observed (S7G and S7H Fig). *tj*^*TS*^*>tra*^*F*^ testes also contained significantly fewer EdU-positive, 4- and 8-cell spermatogonial cysts than *tj*^*TS*^*>+* testes (S7E, S7F and S7K Fig). These results demonstrate that ectopic Tra^F^ in CySCs is deleterious to their differentiation, but alone cannot drive CySCs to assume a follicle-like fate. Taken together with our previous finding that *tra* is downstream of *chinmo* in CySC feminization, we conclude while Tra^F^ induction is important for CySC feminization upon loss of *chinmo*, it is not sufficient.

### Sxl is not required for feminization of *chinmo*-deficient CySCs

Our finding that *chinmo*-deficient CySCs produce *tra*^*F*^ (Fig 5A and 5C) reveals that they possess machinery to splice *tra* pre-mRNA into the female isoform. We considered the possibility that wild type CySCs might be competent to alternatively splice *tra*. However, somatic mis-expression of *UAS*-*traF*^*Δ*^*T2AGFP* in wild type somatic cells did not lead to *tra* alternative splicing, since GFP was absent from the somatic lineage (Fig 5D). Thus, wild type CySCs are intrinsically unable to generate *tra*^*F*^ mRNA, precluding this model.

**Fig 6:**
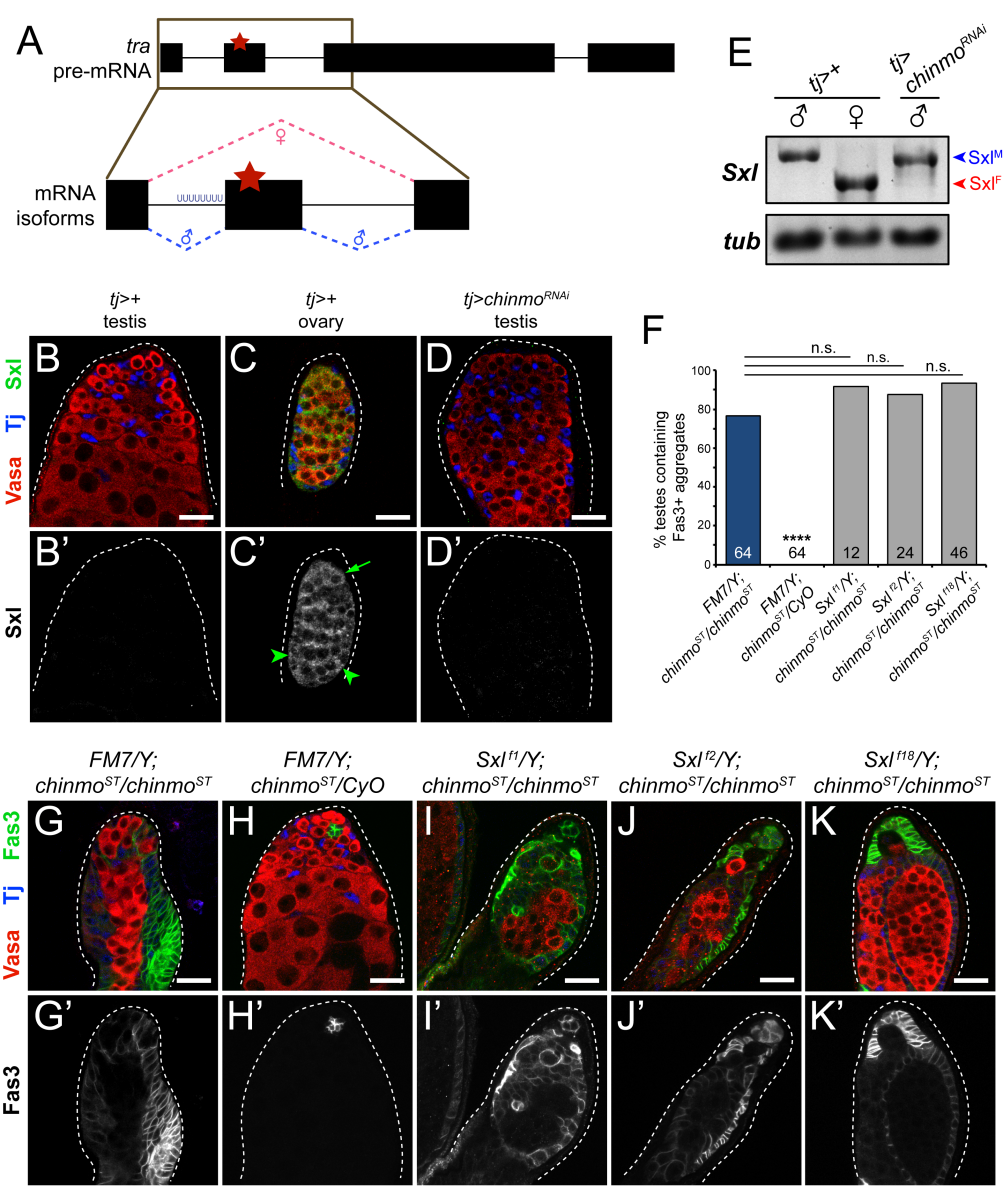
Sxl is not required for feminization of *chinmo*-mutant CySCs. (A) Schematic of *tra* pre-mRNA splicing. The poly(U) tract upstream of exon 2 is bound by the RRM domain of Sxl in females, causing skipping of exon 2. In wild type males, exons 1–4 comprise *tra* mRNA and translation terminates at the early stop codon in exon 2 (red star). Pink dashed lines indicate female-specific alternative splicing and blue dashed lines indicate non-sex-specific default splicing. (B-D) Sxl is not expressed in a control *tj>+* testis (B’) but is expressed in follicle cells (C’, arrowheads) and in an early germ cell (C’, arrow) of a control *tj>+* ovary. Sxl protein is not detected in a *tj>chinmo*^*RNAi*^ testis (D’). (E) Semi-quantitative RT-PCR on *Sxl* in homogenized control *tj>+* testes (left lane), control *tj>+* ovaries (middle lane), and *tj>chinmo*^*RNAi*^ testes (right lane). Control *tj>+* testes express *Sxl*^*M*^ transcripts (blue arrowhead), while control *tj>+* ovaries express *Sxl*^*F*^ transcripts (red arrowhead). *Sxl*^*M*^ is still present and *Sxl*^*F*^ is undetectable in *tj>chinmo*^*RNAi*^ testes (right lane). *Sxl*^*JYR*^ primers were used to differentiate between *Sxl*^*M*^ and *Sxl*^*F*^ mRNA isoforms in this experiment. *α-tubulin* (*tub*) was used as a loading control. (F) Quantification of CySC feminization in *Sxl*; *chinmo*^*ST*^ backgrounds. Sample sizes are indicated within bars. **** denotes p<0.0001 as determined by Fisher’s Exact Test (compared to *FM7/Y*; *chinmo*^*ST*^/*chinmo*^*ST*^). See S1 Table for percentage values. (G-K) Representative images for *Sxl*; *chinmo*^*ST*^ epistasis experiments. Genetic loss of *Sxl* by 3 different alleles–*Sxl* ^*f1*^ (I), *Sxl* ^*f2*^ (J), or *Sxl* ^*f18*^ (K)–does not prevent feminization of *chinmo*^*ST*^*/chinmo*^*ST*^ cyst cells (G’), defined by the accumulation of Fas3-positive aggregates. Control *FM7/Y; chinmo*^*ST*^*/CyO* cyst cells do not feminize (H’). Scale bars = 20 μm.

It follows, then, that one or more factors are ectopically expressed upon loss of *chinmo* that alternatively splice *tra* pre-mRNA into *tra*^*F*^. Since Sxl is required for *tra*^*F*^ production in wild type females (Fig 6A; [12]), we investigated whether Sxl is ectopically expressed in *chinmo*-mutant CySCs. As expected, Sxl protein was absent from wild type testes but was detectable in wild type ovaries (Fig 6B and 6C). Importantly, we did not observe Sxl in *chinmo*-mutant testes (Fig 6D). These results were validated by assessing *Sxl* mRNA isoform abundance in adult gonads. Semi-quantitative RT-PCR demonstrated that control *tj>+* testes express male-specific *Sxl*^*M*^ (Fig 6E, left lane), which contains an early stop codon and encodes no functional protein, while control *tj>+* ovaries express female-specific *Sxl*^*F*^ (Fig 6E, middle lane), which encodes functional Sxl. *tj>chinmo*^*RNAi*^ testes still express *Sxl*^*M*^ (Fig 6E, right lane), consistent with the absence of Sxl protein in these testes (Fig 6D). We also tested whether mis-expression of *chinmo* in female follicle cells could prevent *Sxl* alternative splicing; however, both *tj*^*TS*^*>+* and *tj*^*TS*^*>chinmo* ovaries expressed only the female-specific *Sxl*^*F*^ isoform (S6B Fig).

Furthermore, unlike depletion of *tra*, depletion of *Sxl* in feminizing, *chinmo*-deficient somatic cells did not suppress Fas3 expression or the epithelial organization of somatic cells (Fig 5J, light blue bar; S1 Table). [As expected, somatic depletion of *Sxl* in an otherwise wild type background produced no testis phenotype (S1 Table). We confirmed that the *UAS-Sxl-RNAi* line was effective at knockdown because somatic depletion of *Sxl* in females led to only a rudimentary ovary with 100% penetrance, n = 23.] Consistent with this, none of three distinct mutant alleles of *Sxl* prevented feminization in *chinmo*^*ST*^*/chinmo*^*ST*^ testes (Fig 6F–6K; S1 Table). Taken together, these data support a model where the ectopic *tra* pre-mRNA in *chinmo*-mutant CySCs is alternatively spliced into *tra*^*F*^ via a non-canonical, Sxl-independent mechanism.

### *vir* and *fl(2)d* are upregulated in *chinmo*-deficient CySCs and are required for sex transformation

**Fig 7:**
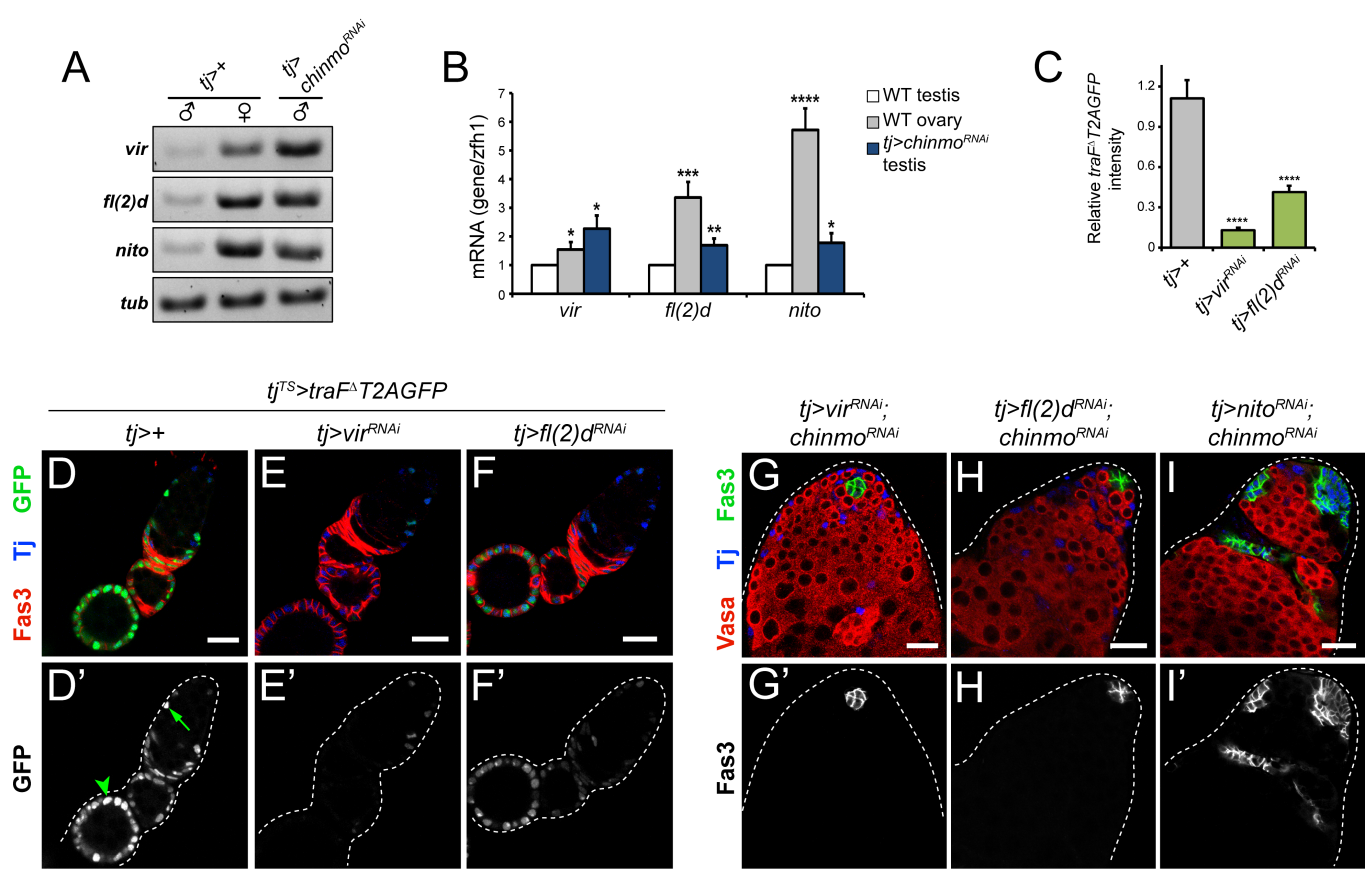
Vir and Fl(2)d, but not Nito, are required for feminization of *chinmo*-deficient CySCs. (A) Semi-quantitative RT-PCR of *vir*, *fl(2)d*, and *nito* in homogenized control *tj>+* testes (left lane), control *tj>+* ovaries (middle lane) and *tj>chinmo*^*RNAi*^ testes (right lane). *vir*, *fl(2)d*, and *nito* are expressed at higher levels in ovaries (middle lane) than in *tj>+* testes (left lane). *vir*, *fl(2)d*, and *nito* are expressed at higher levels in *tj>chinmo*^*RNAi*^ testes (right lane) than in control *tj>+* testes (left lane). *α-tubulin (tub)* was used as a loading control. Flies were aged 9–20 days prior to dissection. (B) qRT-PCR analysis of *vir*, *fl(2)d*, and *nito* in homogenized control *tj>+* testes (white bars), control *tj>+* ovaries (gray bars) and *tj>chinmo*^*RNAi*^ testes (blue bars). *vir*, *fl(2)d*, and *nito* are expressed at significantly higher levels in ovaries and *tj>chinmo*^*RNAi*^ testes compared to control testes. * denotes p<0.05; ** denotes p<0.01; *** denotes p<0.001; **** denotes p<0.0001 as determined by two-tailed Student’s t-test. Error bars represent SEM. Flies were aged 9–20 days prior to dissection. (C) Quantification of GFP levels (synthesized from *UAS-traF*^*Δ*^*T2AGFP* transgene) in *tj>+*, *tj>vir*^*RNAi*^, and *tj>fl(2)d*^*RNAi*^ ovaries represented in D-F. Errors represent SEM. **** denotes p<0.0001 as determined by Student’s t-test. (D-F) Representative images of control *tj>+* (D’), *tj>vir*^*RNAi*^ (E’), and *tj>fl(2)d*^*RNAi*^ (F’) ovaries in a *UAS-traF*^*Δ*^*T2AGFP* background. GFP expressed from *UAS-traF*^*Δ*^*T2AGFP* is detectable in escort cells (D’, arrow) and follicle cells (D’, arrowhead). GFP levels are dramatically reduced upon *vir* or *fl(2)d* depletion (E’ and F’, respectively) compared with wild type follicle cells (D’). Tj (blue) marks escort/follicle cells and Fas3 (red) marks follicle cells. (G-I) Depletion of *nito* (I’) does not reduce Fas3-positive (green) aggregates (a readout for feminization) in *tj>chinmo*^*RNAi*^ testes. By contrast, depletion of *vir* (G’) or *fl(2)d* (H’) in *tj>chinmo*^*RNAi*^ reduces the percentage of testes with these aggregates. Results are quantified in Fig 5J and S1 Table. Vasa (red) marks germ cells and Tj (blue) marks somatic cells. Scale bars = 20 μm.

We next examined a potential role for other candidates with known roles in female-specific alternative splicing of *tra*. We found that *vir*, *fl(2)d*, and *nito* transcripts were 1.5-fold (p<0.05), 3.4-fold (p<0.001), and 5.7-fold (p<0.0001) higher in adult ovaries compared with adult testes, respectively, suggesting sex-biased expression in adult gonads (Fig 7A and 7B). This observation is consistent with ModENCODE RNA-seq data demonstrating that *vir*, *fl(2)d*, and *nito* transcripts are present at very low levels in wild type testes [70]. However, levels of all three transcripts significantly increased (2.3-fold, 1.7-fold, and 1.8-fold for *vir*, *fl(2)d*, and *nito*, respectively) in *tj>chinmo*^*RNAi*^ testes compared with control *tj>+* testes (Fig 7A and 7B; p<0.05 for *vir* and *nito*, p<0.01 for *fl(2)d*). While depleting *vir* or *fl(2)d* had no effect on testis development or spermatogenesis (S8A–S8C Fig), we found that depletion of *vir* in the female somatic gonad caused severe defects in ovary development. *tj>vir*^*RNAi*^ females develop some female reproductive structures and contain an oviduct, but lack ovaries (S8D and S8E Fig). Both *tj>vir*^*RNAi*^ and *tj>fl(2)d*^*RNAi*^ females failed to lay fertilized eggs. To test whether *vir* or *fl(2)d* are necessary for *tra*^*F*^ splicing in adult ovaries, we depleted *vir* or *fl(2)d* in the female somatic gonad using *tj*^*TS*^, rearing flies at the permissive temperature to prevent *vir* or *fl(2)d* knockdown during development. After eclosion, adult females were then reared at the restrictive temperature to allow for *vir* and *fl(2)d* depletion. While wild type follicle cells express GFP produced by *UAS-traF*^*Δ*^*T2AGFP* (Fig 7D), GFP is dramatically reduced in the follicle cells of *tj*^*TS*^*>vir*^*RNAi*^ and *tj*^*TS*^*>fl(2)d*^*RNAi*^ ovaries (Fig 7C, 7E and 7F). These results demonstrate that *vir* and *fl(2)d* are both female-biased in the adult gonad and are required for *tra*^*F*^ alternative splicing in follicle cells.

To test whether *vir* or *fl(2)d* are required for sex transformation upon somatic loss of *chinmo*, we depleted each factor concomitantly with *chinmo* and monitored the frequency of CySC feminization. Depletion of *vir* or *fl(2)d* in *tj>chinmo*^*RNAi*^ testes significantly reduced the percentage of feminized testes (p<0.001 and p<0.0001, respectively) (Fig 7G and 7H; Fig 5J, green and yellow bars, respectively; S1 Table). In contrast, depletion of *nito* did not prevent feminization (Fig 7I; Fig 5J, red bar; S1 Table). As expected, somatic depletion of *vir*, *fl(2)d* or *nito* in an otherwise wild type testis had no effect (S1 Table). We also tested the sufficiency of *fl(2)d* for CySC sex transformation. We found that mis-expression of *fl(2)d* in the adult CySC lineage (*tj*^*TS*^*>fl(2)d*) did not cause Fas3-positive aggregates to accumulate (Fig 5M). Furthermore, the follicle cell marker Cas was not induced in *tj*^*TS*^*>fl(2)d* testes (Fig 5P). Due to the lack of a *UAS-vir* transgenic *Drosophila* line, we were unable to test the sufficiency of *vir* for CySC feminization. Based on these findings, we conclude that Vir and Fl(2)d are epistatic to *chinmo* and are required, but not sufficient, for feminization of *chinmo*-mutant CySCs. Taken together with our previous results, this strongly implicates Vir and Fl(2)d in alternative splicing of the ectopic *tra* pre-mRNA observed in sex-transformed CySCs.

## Discussion

### Chinmo prevents female sex identity in adult male CySCs

**Fig 8:**
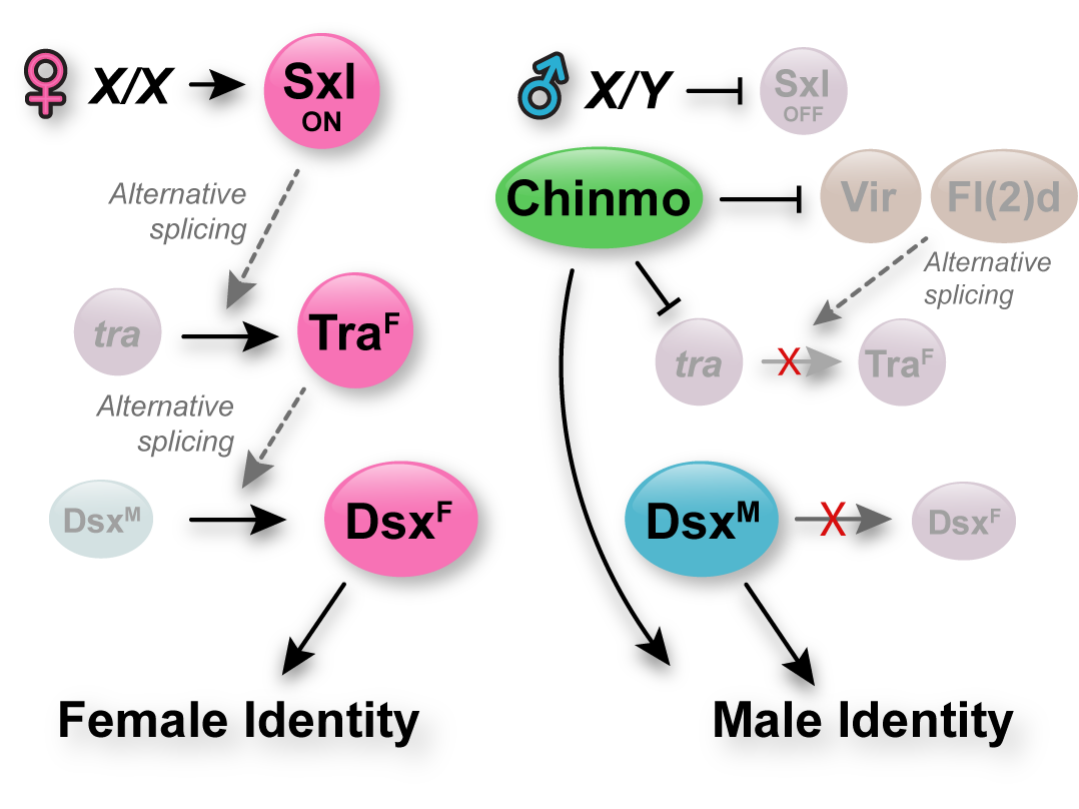
Model for adult somatic sex maintenance in the *Drosophila* somatic gonad. Left: In XX animals, the production of Sxl leads to alternative splicing of *tra* pre-mRNA into *tra*^*F*^. Tra^F^ protein then alternatively splices *dsx* pre-mRNA into *dsx*^*F*^. The Dsx^F^ protein promotes female-specific transcriptional changes. Right: In XY animals, Sxl is not produced. Neither *tra* nor *dsx* pre-mRNA are alternatively spliced, resulting in the production of Dsx^M^ protein, which ensures male-specific transcription of target genes. In addition to the absence of Sxl in XY cells, adult somatic stem cells of the *Drosophila* testis have an extra level of insurance of male sex identity. Chinmo, which is expressed only in male but not female somatic gonadal cells, represses expression of *tra*, *vir* and *fl(2)d* in CySCs. This safeguards male identity by reducing the availability of *tra* pre-mRNA and of factors (i.e., Vir and Fl(2)d)) that can splice it into *tra*^*F*^. Thus, in addition to the canonical sex determination pathway that establishes male and female programs from early development, adult male, sexually dimorphic cells protect their sexual identity by transcriptional repression of *tra* and its splice factors.

Here, we show that that one single factor, Chinmo, preserves the male identity of adult CySCs in the *Drosophila* testis by regulating the levels of canonical sex determinants. We demonstrate that CySCs lacking *chinmo* lose Dsx^M^ expression not by transcriptional loss but rather by alternative splicing of *dsx* pre-mRNA into *dsx*^*F*^. These *chinmo*-mutant CySCs ectopically express Tra^F^ and Dsx^F^, and both factors are required for their feminization. Furthermore, our results demonstrate that *tra* alternative splicing in cyst cells lacking *chinmo* is achieved independently of Sxl. Instead, our work strongly suggests that *tra*^*F*^ production in the absence of *chinmo* is mediated by splicing factors Vir and Fl(2)d. We propose that male sex identity in CySCs is maintained by a two-step mechanism whereby *tra*^*F*^ is negatively regulated at both transcriptional and post-transcriptional levels by Chinmo (Fig 8). In this model, loss of *chinmo* from male somatic stem cells first leads to transcriptional upregulation of *tra* pre-mRNA as well as of *vir* and *fl(2)d*. Then the *tra* pre-mRNA in these cells is spliced into *tra*^*F*^ by the ectopic Vir and Fl(2)d proteins. The ectopic Tra^F^ in *chinmo*-deficient CySCs then splices the *dsx* pre-mRNA into *dsx*^*F*^, resulting in loss of Dsx^M^ and gain of Dsx^F^, and finally induction of target genes usually restricted to follicle cells in the ovary.

### Chinmo regulates levels of *tra*, *vir* and *fl(2)d*

Chinmo has motifs associated with transcriptional repression and its loss clonally is associated with ectopic transcription [54]. One interpretation of our data is that Chinmo directly represses *tra*, *vir*, and *fl(2)d* in male somatic gonadal cells. As the binding site and potential co-factors of Chinmo are not known, future work will be needed to determine whether Chinmo directly regulates expression of these genes. We also note that ~50% of *chinmo*-mutant testes still feminize in the genetic absence of *tra* or *dsx*^*F*^. These latter data indicate that Chinmo regulates male sex identity through another, presumably parallel, mechanism that does not involve canonical sex determinants. However, this *tra*/*dsx*-independent mode of sex maintenance downstream of Chinmo is not characterized and will require the identification of direct Chinmo target genes.

### Regulation of *chinmo* expression in adult CySCs

We previously showed that JAK/STAT signaling promotes *chinmo* in several cell types, including CySCs [54]. Since JAK/STAT signaling is itself sex-biased and restricted to the embryonic male gonad, we presume that activated Stat92E establishes *chinmo* in male somatic gonadal precursors, perhaps as early as they are specified in the embryo [33, 71]. Because loss of Stat92E from CySCs does not result in an apparent sex transformation phenotype [29, 40, 60], we favor the interpretation that Stat92E induces expression of *chinmo* in CySCs but that other sexually biased factors maintain it. One potential candidate is Dsx^M^, which is expressed specifically in early somatic gonads and at the same time when Stat92E activation is occurring in these cells [72]. In fact, multiple Dsx^M^ ChIP-seq peaks were identified in the *chinmo* locus, suggesting potential regulation of *chinmo* by Dsx^M^ [26]. Taken together with our findings, this suggests a potential autoregulatory feedback loop whereby Dsx^M^ preserves its own expression in adult CySCs by maintaining Chinmo expression, which in turn prevents *tra*^*F*^ and *dsx*^*F*^ production.

### Non-canonical mechanisms of sex-specific cell fate and tissue homeostasis

Recent studies on tissue-specific sex maintenance demonstrate that while the Sxl/Tra/Dsx hierarchy is an obligate and linear circuit during embryonic development, at later stages it is more modular than previously appreciated. For example, Sxl can regulate female-biased genes in a *tra*-independent manner [73, 74]. Additionally, Sxl and Tra^F^ regulate body size and gut plasticity independently of the only known Tra^F^ targets, *dsx* and *fru* [3, 4]. We find that negative regulation of the Tra^F^-Dsx^F^ arm of this cascade is required to preserve male sexual identity in CySCs but unexpectedly is independent of Sxl. Because depletion of Vir or Fl(2)d significantly blocks sex transformation and both are required for *tra* alternative splicing in the ovary, our work reveals they can alternatively splice *tra* pre-mRNA even in the absence of Sxl. To the best of our knowledge, this is the first demonstration of Sxl-independent, Tra-dependent feminization. These results raise the broader question of whether other male somatic cells have to safeguard against this novel mechanism. Because recent work has determined that sex maintenance is important in systemic functions regulated by adipose tissue and intestinal stem cells [3, 4], it will be important to determine whether Chinmo represses *tra*^*F*^ in these settings. Finally, since the transcriptional output of the sex determination pathway is conserved from *Drosophila* (Dsx) to mammals (DMRT1), it is possible that transcriptional regulation of sex determinants plays a similar role in adult tissue homeostasis and fertility in higher organisms.

## Materials and methods

### Fly stocks and husbandry

The following fly stocks were used and are described in FlyBase: Oregon^R^; *yw; tj-gal4*; *tub-gal80*^*TS*^; *dsx-gal4; dsx-gal*^*Δ2*^*; GMR40A05-gal4; dsx*^*MI03050-GFSTF*.*1*^*; dsx*^*1*^; *dsx*^*D*^; *chinmo*^*ST*^; *tra*^*1*^; *Df(3L)st-j7*; *UAS-GFP*^*nls*^; *UAS-dcr2*; *UAS-chinmo*^*RNAi*^ *(HMS00036)*; *UAS-tra*^*RNAi*^ *(HMS02830)*; *UAS-tra*^*F*^; *UAS-3xHAfl(2)d*; *UAS-dsx*^*F*^*; UAS-5’UTR-chinmo-3’UTR; UAS-Sxl*^*RNAi*^ *(HMS00609)*; *Sxl*^*f1*^*; Sxl*^*f2*^*; Sxl*^*f18*^*; UAS-vir*^*RNAi*^ *(HMC03908)*; *UAS-fl(2)d*^*RNAi*^ *(HMC03833)*; *UAS-nito*^*RNAi*^ *(HMS00166)*.

For RNAi-mediated depletion of *chinmo*, *Sxl*, *tra*, *vir*, *fl(2)d*, and *nito*, flies were reared at an ambient temperature (21°C). Adult males were collected twice a week and aged at 29°C to increase Gal4 activity. For temporal control of gene expression, *tj-gal4*, *tub-gal80*^*TS*^ virgins were crossed to *UAS-tra*^*F*^ or *UAS-3xHAfl(2)d* males and progeny were reared at the permissive temperature (18°C) to prevent *tra*^*F*^ or *fl(2)d* mis-expression during embryonic, larval, and pupal development. Adult males of the correct genotype were collected twice a week and shifted to the restrictive temperature (29°C) to inactivate Gal80.

### Generation of *UAS-traF*^*Δ*^*T2AGFP*

In this transgene, most of the third exon of *tra* is replaced by the coding sequences for self-cleaving T2A peptide and GFP. Specifically, the coding sequences of T2A and GFP were cloned in frame immediately downstream of the 26^th^ nucleotide (nt) of *tra* exon 3 and immediately upstream of the last 18 nt of this exon. PCR was performed with Q5 high-fidelity polymerase from New England Biolabs (M0491S). The PCR product was digested with EcoRI and XhoI before cloning into the *pUASTattb* vector [75]. The construct was verified by sequencing, and a transgenic line was established through ΦC-31 integrase mediated transformation (Bestgene, attP site VK05, BDSC#9725).

### Antibodies

The following primary antibodies were used: rat anti-Chinmo (1:1000; gift of N. Sokol, Indiana University, IN, USA), goat anti-Vasa (1:50, dC-13, Santa Cruz), rabbit anti-Vasa (1:1500; gift of R. Lehmann, Skirball Institute/NYU School of Medicine, NY, USA), guinea pig anti-Tj (1:5000; gift of D. Godt, University of Toronto, ON, Canada), rabbit anti-Zfh1 (1:5000; gift of R. Lehmann), mouse anti-Fasciclin-3 (1:50; Developmental Studies Hybridoma Bank (DSHB)), mouse anti-Eya (1:20; DSHB), rat anti-Dsx^M^ (1:200; gift of B. Oliver, National Institutes of Health, MD, USA), rat anti-Dsx^C^ (1:50; gift of M. Arbeitman, Florida State University, FL, USA), rabbit anti-Castor (1:50; gift of W. Odenwald, National Institutes of Health, MD, USA), mouse anti-α-spectrin (1:20, DSHB), mouse anti-Sxl^M18^ (1:5; DSHB), rabbit anti-GFP (1:500; Invitrogen). Secondary antisera used were all raised in donkey (Jackson ImmunoResearch).

### Immunofluorescence

Testes and ovaries were dissected in 1x PBS and fixed in 4% paraformaldehyde in 1x PBS for 30 minutes at room temperature (RT). Fixed tissue was washed twice at RT in 0.5% PBST (1x PBS with 0.5% Triton X-100) and blocked in PBTB (1x PBS, 0.2% Triton X-100, 1% BSA) for 1 hour at RT or overnight at 4°C. Primary antibodies were incubated overnight at 4°C and washed off twice at RT in PBTB. Secondary antibodies were incubated for 2 hours at RT in the dark and washed off twice in 0.2% PBST (1x PBS with 0.2% Triton X-100). Tissue was mounted in Vectashield Medium (Vector Laboratories) prior to confocal analysis, and confocal images were captured using a Zeiss LSM 510 confocal microscope, 63x objective.

### DIC microscopy on whole ovaries

DIC images of adult female reproductive structures (at 5x) were obtained using a Zeiss Axioplan microscope with a Retiga Evi (QImaging) digital camera and QCapture Pro 6.0 software.

### Immunofluorescence using anti-Dsx^C^

Testes and ovaries were dissected in 1x PBS and fixed in 20% EM-grade paraformaldehyde (Electron Microscopy Sciences) in 1x PBS for 20 minutes at RT. Fixed tissue was washed 3 times for 15 minutes each in TNT (0.1M Tris-HCl, 0.3M NaCl, 0.05% Tween-20) and blocked using Image-iT FX Signal Enhancer (ThermoFisher) for 30 minutes at RT, then washed 3 times for 15 minutes each in TNT. Primary anti-Dsx^C^ was incubated overnight at 4°C. After anti-Dsx^C^ incubation, tissue was blocked in PBTB for 1 hour and then treated with anti-Vasa and anti-Tj. Primary antibodies were washed twice for 15 minutes each in PBTB, then secondary antibodies were incubated overnight at 4°C in PBTB. Finally, the Dsx^C^ signal was amplified by TSA (see below) and testes were mounted in Vectashield prior to analysis.

### Tyramide signal amplification (TSA)

TSA (Perkin Elmer) was performed to amplify Dsx^M^ and Dsx^C^ signals. HRP anti-rat (Jackson ImmunoResearch) was used as a secondary antibody and the tertiary Cy3-conjugated tyramide reaction was performed per the manufacturer’s instructions.

### CySC purification by fluorescence-activated cell sorting (FACS)

To purify CySCs and early cyst cells, the somatic cell lineage was labeled using *tj-gal4* to drive *UAS-GFP*^*nls*^ expression. Testes were dissociated in trypsin/collagenase for 15 minutes and the cell suspension was passed through 70μm filters (Falcon). GFP-expressing somatic cells were purified from the resulting filtrate by FACS using a Sony SY3200 highly automated parallel sorting (HAPS) cell sorter into TRIzol LS (ThermoFisher), and RNA was extracted according to the manufacturer’s instructions. Post-sort purity of samples was confirmed by immunocytochemistry and the absence of Vasa-positive germ cells.

### 5-ethynyl-2’-deoxyuridine (EdU)-labeling of adult testes

EdU-labeling of testes was performed using the Click-iT EdU Alexa Fluor 647 Imaging Kit (ThermoFisher). Testes were dissected in S2 cell culture medium (Life Technologies) then incubated in 10 μM EdU for 30 minutes. Testes were then fixed, washed, and stained as described above. The cycloaddition reaction was performed per the manufacturer’s instructions. Testes were mounted in Vectashield prior to confocal analysis.

### Quantitative and semi-quantitative RT-PCR

To detect mRNA levels of canonical sex determinants by PCR, whole ovaries (n = 5–10) or whole testes (n = 55–200) were isolated and homogenized into TRIzol (ThermoFisher). RNA was extracted and DNase-treated (Ambion) per the manufacturer’s instructions. Reverse transcription was performed using Maxima reverse transcriptase (ThermoFisher) according to the manufacturer’s instructions and 1–2 μg of RNA as template. qRT-PCR was performed using SYBR Green PCR Master Mix (ThermoFisher) and a Biorad CFX96 Real-Time PCR Machine. Semi-quantitative RT-PCR was performed on a Biorad iCycler. Because the proportion of somatic cells is significantly increased in *tj>chinmo*^*RNAi*^ testes compared to *tj>+* controls, the qRT-PCR values were normalized first to *tubulin* and second to *zfh1*, an early somatic marker.

### Primers

*total tra*: fwd-GAGCCCGCATCGGTATAATC; rev-GACGTGGTAGCCTTTGGTATC

*tra*^*F*^: fwd-AACCCAGCATCGAGATTCC; rev-CGAACCTCGTCTGCAAAGTA

*dsx*^*C*^: fwd-GAAAGAACGGCGCCAAT; rev-GGCGTCTGCGTCCTTTAATA

*dsx*^*M*^: fwd-GAGCTGATGCCACTCATGTAT; rev-CTGGGCTACAGTGCGATTTA

*dsx*^*F*^: fwd-GAATGAGTACTCCCGTCAACAT; rev-GGGCAAAGTAGTATTCGTTACTCTA

*rpl15*: fwd-AGGATGCACTTATGGCAAGC; rev-GCGCAATCCAATACGAGTTC

*α-tub84b*: fwd-CAACCAGATGGTCAAGTGCG; rev-ACGTCCTTGGGCACAACATC

*β-tub56d*: fwd-CTCAGTGCTCGATGTTGTCC; rev-GCCAAGGGAGTGTGTGAGTT

*Sxl*^*JYR*^: fwd-ACACAAGAAAGTTGAACAGAGG; rev-CATTCCGGATGGCAGAGAATGG

*Sxl*^*EM*^: fwd-CGCTGCGAGTCCATTTCC; rev-GTGGTTATCCCCCATATGGC

*vir*: fwd-CATGAGGAAGTGACGGACATC; rev-GGAAAGTCTGCCTGGACTCG

*fl(2)d*: fwd-GGCCAACAAGGAGCAAGAA; rev-CGCTCGAACAGGAGATTGAC

*nito*: fwd-GGTGTACAAGTCCACAACCAGA; rev-CGACGGTGATCCAAAGGAA

### Fertility assays

The fertility of adult males was assayed by mating individual males with two wild type (Oregon^R^) virgin females (between 5–10 days old) for 48 hours at 25°C. After a 2-day mating period, males were recovered and preserved for subsequent matings using fresh virgin Oregon^R^ females. Fertility was scored by counting the number of F1 offspring produced by each individual cross and reported as the average number of F1 offspring for each genotype.

### Statistical analysis

Statistical parameters for each experiment are reported in the figure legends. Data were analyzed using Microsoft Excel and are reported to be statistically significant when p<0.05 by the appropriate statistical test. For qRT-PCR data, significance was determined by two-tailed Student’s t-test. For fertility assays and cyst cell quantifications, significance was determined using single-factor ANOVA. For rescue of CySC feminization (Fas3-positive aggregates), significance was determined using Fisher’s Exact Test.

## Supporting information

S1 Table Quantification of testes with Fas3-positive somatic aggregates (referred to as “feminized”). Data are presented as the percentage of testes with Fas-3-positive aggregates in testes of the indicated genotypes from the total number of testes examined.
(DOCX)

S1 Fig Loss of chinmo in the CySC lineage causes sex transformation and loss of the germline. (A-C) A transcriptional reporter for *slow border cells* (*slbo-GFP*) is not expressed in a wild type testis (A’, arrowhead). *slbo-GFP* (green) is expressed in mature follicle cells (B’, arrowhead). *slbo-GFP* is ectopically expressed in the CySC lineage upon loss of *chinmo* (C’, arrowhead). Time point is 11 days post-eclosion. Tj (blue) marks cyst cells. Vasa (red) marks the germline.
(D) Semi-quantitative RT-PCR on *Yp1* using RNA extracts from homogenized control *tj>+* testes (left lane), control *tj>+* ovaries (middle lane), and *tj>chinmo^RNAi^* testes (right lane). *Yp1* is expressed in control *tj>+* ovaries (middle lane), but not in control *tj>+* testes (left lane). In *tj>chinmo^RNAi^* testes (right lane), *Yp1* is expressed. *α-tubulin* (*tub*) was used as a loading control. Timepoint is 9–14 days post-eclosion.
(E-F) Loss of *chinmo* in male niche cells using *upd-gal4*, *gal80*^*TS*^ (*upd*^*TS*^) causes no overt defects in testis development or spermatogenesis. Time point is 8 days post-eclosion. TOPRO (blue) marks DNA. Fas3 (green) marks niche cells.
(G-H) Representative images of agametic *tj>chinmo^RNAi^* testes at 7 days post-eclosion. Fas3-positive somatic aggregates (green) fill the apex of the testis, which is devoid of Vasa-positive (red) germ cells. Zfh1 (blue) marks somatic cells.
(I-K) Expression of Chinmo in adult gonads. Chinmo is expressed in the CySC lineage of the adult testis (I’, arrowheads) but is absent from follicle cells in the adult ovary (J’, arrowheads). Upon chinmo depletion in the testis (*tj>chinmo^RNAi^*), Chinmo protein is lost from feminizing cyst cells (K’, arrowheads). The remaining Chinmo protein observed in K’ represents Chinmo expression in the male germline. Vasa (red) marks germ cells and Tj (blue) marks somatic cells.
Scale bars = 20 μm.
(TIF)

S2 Fig Three different *dsx* transcriptional reporters show variable expression in adult gonads. (A-B) Expression of *dsx-gal4*^*Δ2*^ in adult gonads. In the testis, *dsx-gal4*^*Δ2*^ is expressed in the entire CySC lineage (A). In the ovary, *dsx-gal4*^*Δ2*^ is expressed in escort cells, but not follicle cells (B).
(C-D) Expression of *GMR40A05-gal4* in adult gonads. In the testis, *GMR40A05-gal4* is expressed in the entire CySC lineage (C). In the ovary, *GMR40A05-gal4* is expressed in escort cells, but not follicle cells (D).
(E-F) Expression of *dsx*^*MI03050-GFSTF*.*1*^ in adult gonads. In the testis, *dsx*^*MI03050-GFSTF*.*1*^ is expressed weakly in the CySC lineage (E) and is undetectable in adult ovaries (F).
Fas3 (red) marks testicular niche cells and ovarian follicle cells. Tj (blue) marks somatic cells in both gonads. Time point for all adults is 5 days post eclosion. Scale bars = 20 μm.
(TIF)

S3 Fig Dsx^C^ antibody detects Dsx^F^ protein. Immunostaining of *tj>dsx*^*F*^ ovaries reveals that Dsx^F^ protein is detectable by Dsx^C^ antibody (magenta). Tj (green) marks somatic cells. Scale bars = 20 μm.
(TIF)

S4 Fig Blocking Dsx^F^ or Tra^F^ production genetically in females causes masculinization of the soma. (A-D) Blocking *dsx*^*F*^ production using the *dsx*^*D*^*/dsx*^*1*^ heteroallelic combination masculinizes the soma of XX animals. Chromosomal sex of flies was determined based on inheritance of X-linked traits (eye color, *w*; cuticle color, *y*). Genotype for A (XX animal) is *yw/y*^*+*^*w*^*+*^*; chinmo*^*ST*^*/chinmo*^*ST*^*; dsx*^*1*^*/TM2*; for B (XX animal) is *yw/y*^*+*^*w*^*+*^*; chinmo*^*ST*^*/chinmo*^*ST*^*; dsx*^*D*^*/dsx*^*1*^; for C (XY animal) is *yw/Y; chinmo*^*ST*^*/chinmo*^*ST*^*; dsx*^*1*^*/TM2*; for D (XY animal) is *yw/Y; chinmo*^*ST*^*/chinmo*^*ST*^*; dsx*^*D*^*/dsx*^*1*^.
(E-H) Blocking *tra*^*F*^ production using *tra*^*1*^*/Df(3L)st-j7*, *Ki*^*1*^ masculinizes the soma of XX animals. Chromosomal sex of flies was determined based on inheritance of X-linked traits (eye color, *w*). Genotype for E (XX animal) is *w/w*^*+*^*; chinmo*^*ST*^*/chinmo*^*ST*^*; tra*^*1*^*/TM6B*, *Tb*; for F (XX animal) is *w/w*^*+*^*; chinmo*^*ST*^*/chinmo*^*ST*^*; tra*^*1*^*/Df(3L)st-j7*, *Ki*^*1*^; for G (XY animal) is *w/Y; chinmo*^*ST*^*/chinmo*^*ST*^*; tra*^*1*^*/TM6B*, *Tb*; for H (XY animal) is *w/Y; chinmo*^*ST*^*/chinmo*^*ST*^*; tra*^*1*^*/Df(3L)st-j7*, *Ki*^*1*^.
(TIF)

S5 Fig Diagram of *tra* pre-mRNA and *UAS-traF^Δ^T2AGFP*. In the transgene, most of the third exon of *tra* is replaced with self-cleaving T2A peptide and GFP, followed by a poly-adenylation signal (pA). Black shaded regions indicate exons. Red star indicates early stop codon in exon 2. Pink dashed lines indicate female-specific alternative splicing, and blue dashed lines indicate non-sex-specific default splicing.
(TIF)

S6 Fig Chinmo mis-expression in ovaries leads to reduced *tra^F^* and *dsx^F^* levels. (A) qRT-PCR analysis of homogenized ovaries demonstrates that mis-expression of *chinmo* in follicle cells leads to decreased levels of total *tra*, *tra*^*F*^, and *dsx*^*F*^. Lower transcript levels were not due to a change in the relative abundance of somatic cells, as *zfh1* levels were unaffected in *tj*^*TS*^*>chinmo* ovaries. The values were normalized to *tubulin*. Data are presented as the mean of three biological replicates. *** denotes p<0.001 as determined by two-tailed Student’s t-test. Error bars represent SEM.
(B) Semi-quantitative RT-PCR on RNA extracts from 5 male or 5 female larvae (first two lanes), *tj*^*TS*^*>+* adult ovaries (third lane), and *tj*^*TS*^*>chinmo* adult ovaries (last lane). RNA from male larvae express *Sxl*^*M*^ (first lane), while RNA from female larvae express *Sxl*^*F*^ (second lane). Both *tj*^*TS*^*>+* (third lane) and *tj*^*TS*^*>chinmo* (last lane) ovaries express *Sxl*^*F*^ exclusively. *Sxl*^*EM*^ primers were used to differentiate between *Sxl*^*M*^ and *Sxl*^*F*^ mRNA isoforms in this experiment. *α-tubulin* (*tub*) was used as a loading control.
(TIF)

S7 Fig Tra^F^ is necessary but not sufficient for CySC feminization. (A-B) Zfh1 (blue) expression in *tj*^*TS*^*>+* (A) versus *tj*^*TS*^*>tra*^*F*^ (B) testes. A and B represent single Z slices; A’ and B’ show maximal Z-projections (Z-max) of Zfh1-expressing cells in the entire confocal stack. Fas3 (green) marks the niche.
(C-D) Tj (blue) expression in *tj*^*TS*^*>+* (C) versus *tj*^*TS*^*>tra*^*F*^ (D) testes. C and D represent single Z slices; C’ and D’ show Z-max projections of Tj-expressing cells.
(E-F) EdU (blue)-labeled *tj*^*TS*^*>+* (E) and *tj*^*TS*^*>tra*^*F*^ (F) testes. EdU-positive spermatogonial cysts are outlined. Tj (green) marks cyst cells. Arrowheads (E’) point to EdU-positive CySCs. Arrows (F’) point to EdU-positive differentiating cyst cells away from the niche. Asterisk marks the niche.
(G-H) Visualization of germ cell stages in *tj*^*TS*^*>+* (G) and *tj*^*TS*^*>tra*^*F*^ (H) testes. α-spectrin (green) marks fusomes, which are dot- and dumbbell-shaped in early germ cells (G’, arrowheads) and become branched in later differentiating spermatogonia (G”, arrows). Note that the niche is not in the plane in G’. Tj (blue) marks cyst cells. Arrowheads in H’ indicate spermatogonia away from the niche that have dot and dumbbell shape fusomes in *tj*^*TS*^*>tra*^*F*^ testes. Asterisk marks the niche.
(I-J) Quantification of Zfh1-expressing (I) and Tj-expressing (J) cells in *tj*^*TS*^*>+* (gray bars) versus *tj*^*TS*^*>tra*^*F*^ (green bars) testes. *tj*^*TS*^*>tra*^*F*^ testes contain significantly more Zfh1-expressing and Tj-expressing somatic cells than *tj*^*TS*^*>+* testes, as determined by single-factor ANOVA.
(K) Quantification of EdU-positive germ cells upon somatic *tra*^*F*^ mis-expression. *tj*^*TS*^*>tra*^*F*^ testes contain significantly fewer EdU-positive 4-cell and 8-cell spermatogonia than *tj*^*TS*^*>*+ testes.
For quantifications, * denotes p<0.05; ** denotes p<0.01; *** denotes p<0.001; **** denotes p<0.0001 as determined by single-factor ANOVA.
Quantification data are presented as mean ± SEM.
Vasa (red) marks the germline in A-H. Scale bars = 20 μm.
(TIF)

S8 Fig Loss of *vir* and *fl(2)d* causes defects in the ovary but not the testis. (A-C) *tj>vir*^*RNAi*^ (B) and *tj>fl(2)d*^*RNAi*^ (C) testes resemble control *tj>+* (A) testes, showing no overt defects in testis development or spermatogenesis. Vasa (red) marks the germline, Tj (blue) marks somatic cells, and Fas3 (green) marks niche cells. Scale bars = 20 μm.
(D-E) Reproductive structures in adult *tj>+* (D) and *tj>vir*^*RNAi*^ (E) females. Ovaries (D, brackets) and accessory structures like spermathecae (SP) (D, arrows) can be observed in *tj>+* females. Ovaries, but not somatic accessory structures like SP and oviduct, fail to develop in females lacking *vir* in the somatic gonad (E, arrows).
(TIF)
