# Supplementary material for: Chinmo prevents transformer alternative splicing to maintain male sex identity
Source: PLoS Genet. 2018 Feb 1;14(2):e1007203. doi: 10.1371/journal.pgen.1007203 (PMC5811060; doi:10.1371/journal.pgen.1007203)
Supplement: S1 Table — Data are presented as the percentage of testes with Fas-3-positive aggregates in testes of the indicated genotypes from the total number of testes examined. (DOCX) [file pgen.1007203.s001.docx]

**Table S1. Quantification of testes with Fas3-positive somatic aggregates (referred to as “feminized”)**

| ***tj-gal4, uas-dcr2* with:** | **% feminized (n)** |
| --- | --- |
| *gfp; chinmo^RNAi^* | 97 (41) |
| *tra^RNAi^* | 0 (18) |
| *tra^RNAi^; chinmo^RNAi^* | 48 (52) |
| *Sxl^RNAi^* | 0 (25) |
| *Sxl^RNAi^; chinmo^RNAi^* | 94 (33) |
| *vir^RNAi^* | 0 (32) |
| *vir^RNAi^; chinmo^RNAi^* | 65 (46) |
| *fl(2)d^RNAi^* | 0 (25) |
| *fl(2)d^RNAi^; chinmo^RNAi^* | 49 (37) |
| *nito^RNAi^* | 0 (14) |
| *nito^RNAi^; chinmo^RNAi^* | 100 (28) |
|  |  |
| **Genetic “rescue” genotypes** | **% feminized (n)** |
| *chinmo^ST/ST^; TM2/TM6B* | 100 (14) |
| *chinmo^ST^/CyO; dsx^D^/dsx^1^* | 0 (20) |
| *chinmo^ST/ST^; dsx^D^/dsx^1^* | 57 (61) |
| *chinmo^ST/ST^; dsx^D^/TM6B* | 81 (21) |
| *chinmo^ST/ST^; dsx^1^/TM6B* | 92 (37) |
| *chinmo^ST/ST^; tra^1^/Df(3L)st-j7* | 61 (28) |
| *chinmo^ST/ST^; tra^1^/TM6B* | 96 (26) |
| *chinmo^ST/ST^; Df(3L)st-j7/TM6B* | 95 (21) |
| *FM7/Y;chinmo^ST/ST^* | 76 (64) |
| *FM7/Y;chinmo^ST^/CyO* | 0 (64) |
| *Sxl ^f1^/Y;chinmo^ST/ST^* | 92 (12) |
| *Sxl ^f2^/Y;chinmo^ST/ST^* | 88 (24) |
| *Sxl ^f18^/Y;chinmo^ST/ST^* | 93 (46) |
|  |  |
