# Supplementary figures and images for: Chinmo prevents transformer alternative splicing to maintain male sex identity
Source: PLoS Genet. 2018 Feb 1;14(2):e1007203. doi: 10.1371/journal.pgen.1007203 (PMC5811060; doi:10.1371/journal.pgen.1007203)

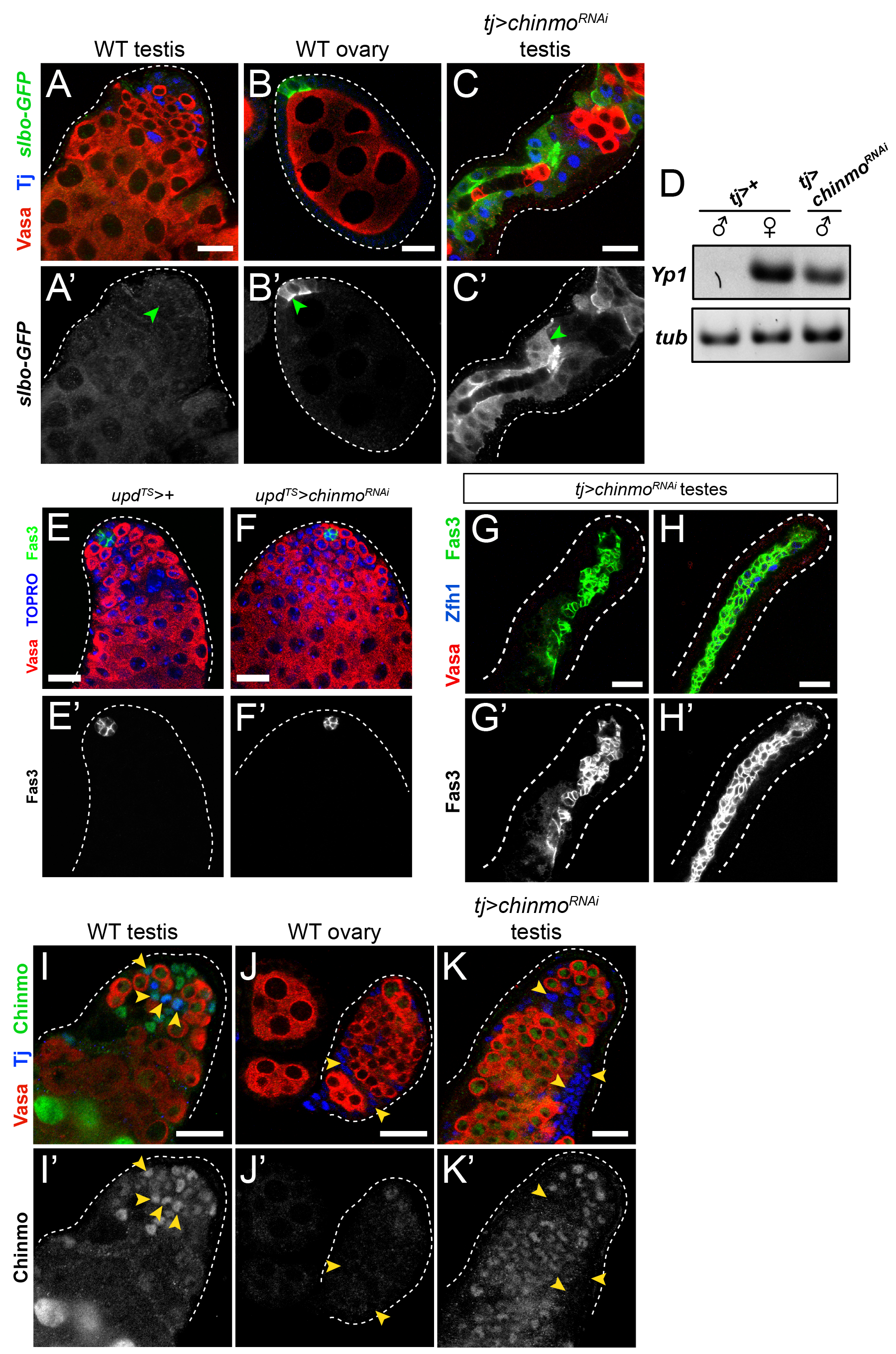

Supplement: S1 Fig — (A-C) A transcriptional reporter for slow border cells (slbo-GFP) is not expressed in a wild type testis (A’, arrowhead). slbo-GFP (green) is expressed in mature follicle cells (B’, arrowhead). slbo-GFP is ectopically expressed in the CySC lineage upon loss of chinmo (C’, arrowhead). Time point is 11 days post-eclosion. Tj (blue) marks cyst cells. Vasa (red) marks the germline. (D) Semi-quantitative RT-PCR on Yp1 using RNA extracts from homogenized control tj>+ testes (left lane), control tj>+ ovaries (middle lane), and tj>chinmoRNAi testes (right lane). Yp1 is expressed in control tj>+ ovaries (middle lane), but not in control tj>+ testes (left lane). In tj>chinmoRNAi testes (right lane), Yp1 is expressed. α-tubulin (tub) was used as a loading control. Timepoint is 9–14 days post-eclosion. (E-F) Loss of chinmo in male niche cells using upd-gal4, gal80TS (updTS) causes no overt defects in testis development or spermatogenesis. Time point is 8 days post-eclosion. TOPRO (blue) marks DNA. Fas3 (green) marks niche cells. (G-H) Representative images of agametic tj>chinmoRNAi testes at 7 days post-eclosion. Fas3-positive somatic aggregates (green) fill the apex of the testis, which is devoid of Vasa-positive (red) germ cells. Zfh1 (blue) marks somatic cells. (I-K) Expression of Chinmo in adult gonads. Chinmo is expressed in the CySC lineage of the adult testis (I’, arrowheads) but is absent from follicle cells in the adult ovary (J’, arrowheads). Upon chinmo depletion in the testis (tj>chinmoRNAi), Chinmo protein is lost from feminizing cyst cells (K’, arrowheads). The remaining Chinmo protein observed in K’ represents Chinmo expression in the male germline. Vasa (red) marks germ cells and Tj (blue) marks somatic cells. Scale bars = 20 μm. (TIF) [file pgen.1007203.s002.tif]

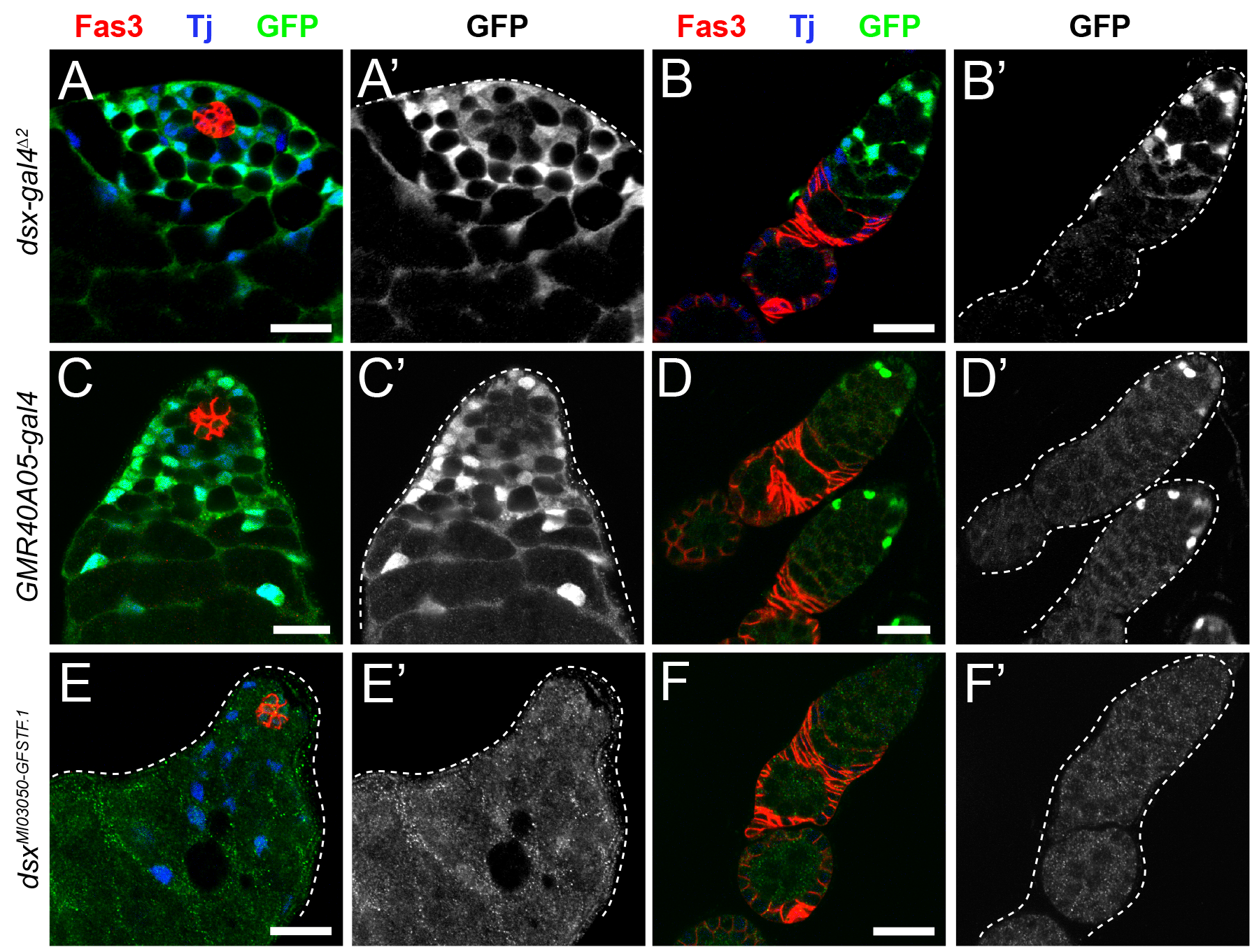

Supplement: S2 Fig — (A-B) Expression of dsx-gal4Δ2 in adult gonads. In the testis, dsx-gal4Δ2 is expressed in the entire CySC lineage (A). In the ovary, dsx-gal4Δ2 is expressed in escort cells, but not follicle cells (B). (C-D) Expression of GMR40A05-gal4 in adult gonads. In the testis, GMR40A05-gal4 is expressed in the entire CySC lineage (C). In the ovary, GMR40A05-gal4 is expressed in escort cells, but not follicle cells (D). (E-F) Expression of dsxMI03050-GFSTF.1 in adult gonads. In the testis, dsxMI03050-GFSTF.1 is expressed weakly in the CySC lineage (E) and is undetectable in adult ovaries (F). Fas3 (red) marks testicular niche cells and ovarian follicle cells. Tj (blue) marks somatic cells in both gonads. Time point for all adults is 5 days post eclosion. Scale bars = 20 μm. (TIF) [file pgen.1007203.s003.tif]

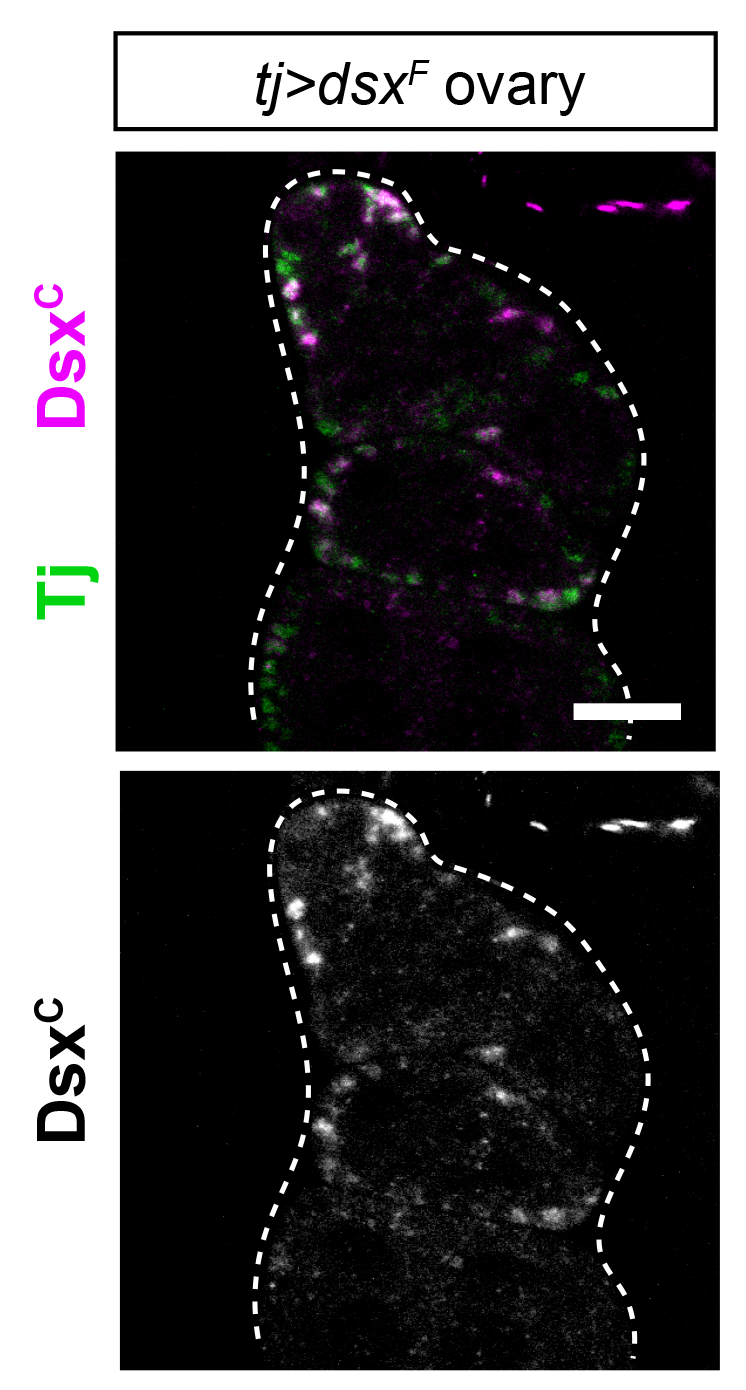

Supplement: S3 Fig — Immunostaining of tj>dsxF ovaries reveals that DsxF protein is detectable by DsxC antibody (magenta). Tj (green) marks somatic cells. Scale bars = 20 μm. (TIF) [file pgen.1007203.s004.tif]

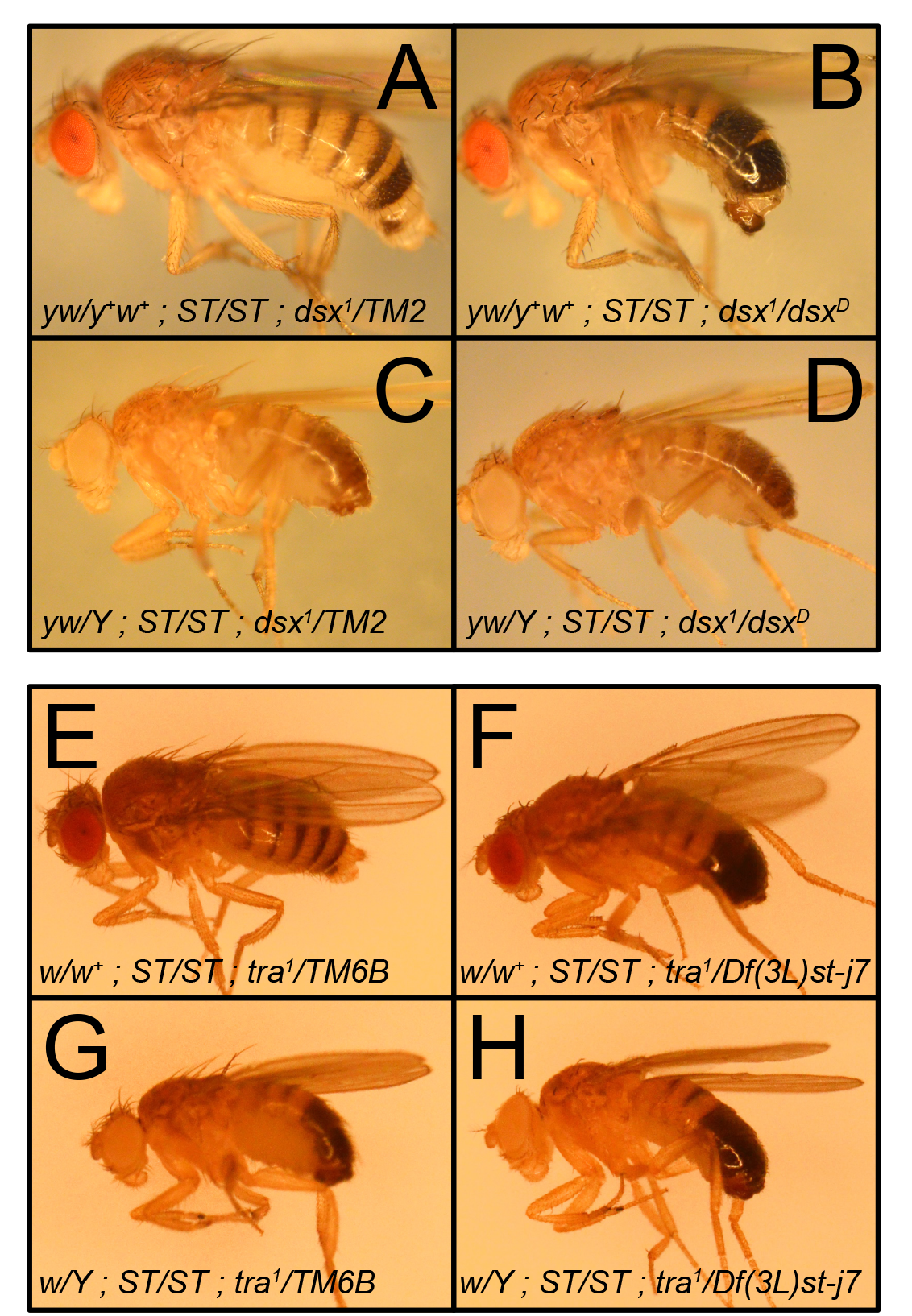

Supplement: S4 Fig — (A-D) Blocking dsxF production using the dsxD/dsx1 heteroallelic combination masculinizes the soma of XX animals. Chromosomal sex of flies was determined based on inheritance of X-linked traits (eye color, w; cuticle color, y). Genotype for A (XX animal) is yw/y+w+; chinmoST/chinmoST; dsx1/TM2; for B (XX animal) is yw/y+w+; chinmoST/chinmoST; dsxD/dsx1; for C (XY animal) is yw/Y; chinmoST/chinmoST; dsx1/TM2; for D (XY animal) is yw/Y; chinmoST/chinmoST; dsxD/dsx1. (E-H) Blocking traF production using tra1/Df(3L)st-j7, Ki1 masculinizes the soma of XX animals. Chromosomal sex of flies was determined based on inheritance of X-linked traits (eye color, w). Genotype for E (XX animal) is w/w+; chinmoST/chinmoST; tra1/TM6B, Tb; for F (XX animal) is w/w+; chinmoST/chinmoST; tra1/Df(3L)st-j7, Ki1; for G (XY animal) is w/Y; chinmoST/chinmoST; tra1/TM6B, Tb; for H (XY animal) is w/Y; chinmoST/chinmoST; tra1/Df(3L)st-j7, Ki1. (TIF) [file pgen.1007203.s005.tif]

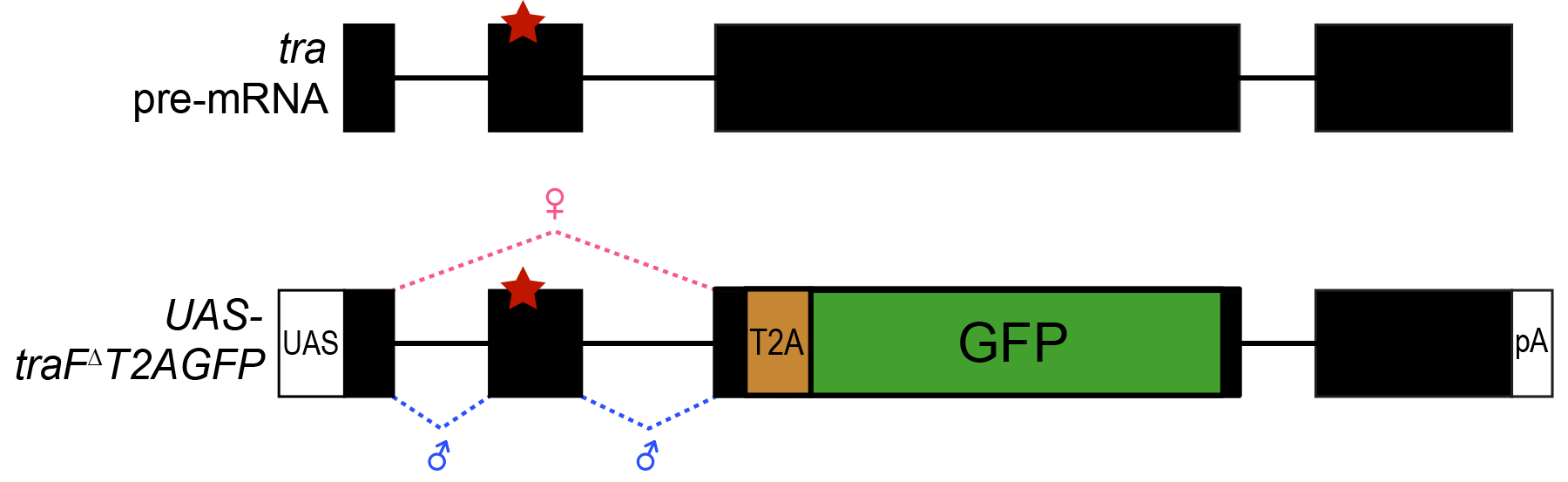

Supplement: S5 Fig — In the transgene, most of the third exon of tra is replaced with self-cleaving T2A peptide and GFP, followed by a poly-adenylation signal (pA). Black shaded regions indicate exons. Red star indicates early stop codon in exon 2. Pink dashed lines indicate female-specific alternative splicing, and blue dashed lines indicate non-sex-specific default splicing. (TIF) [file pgen.1007203.s006.tif]

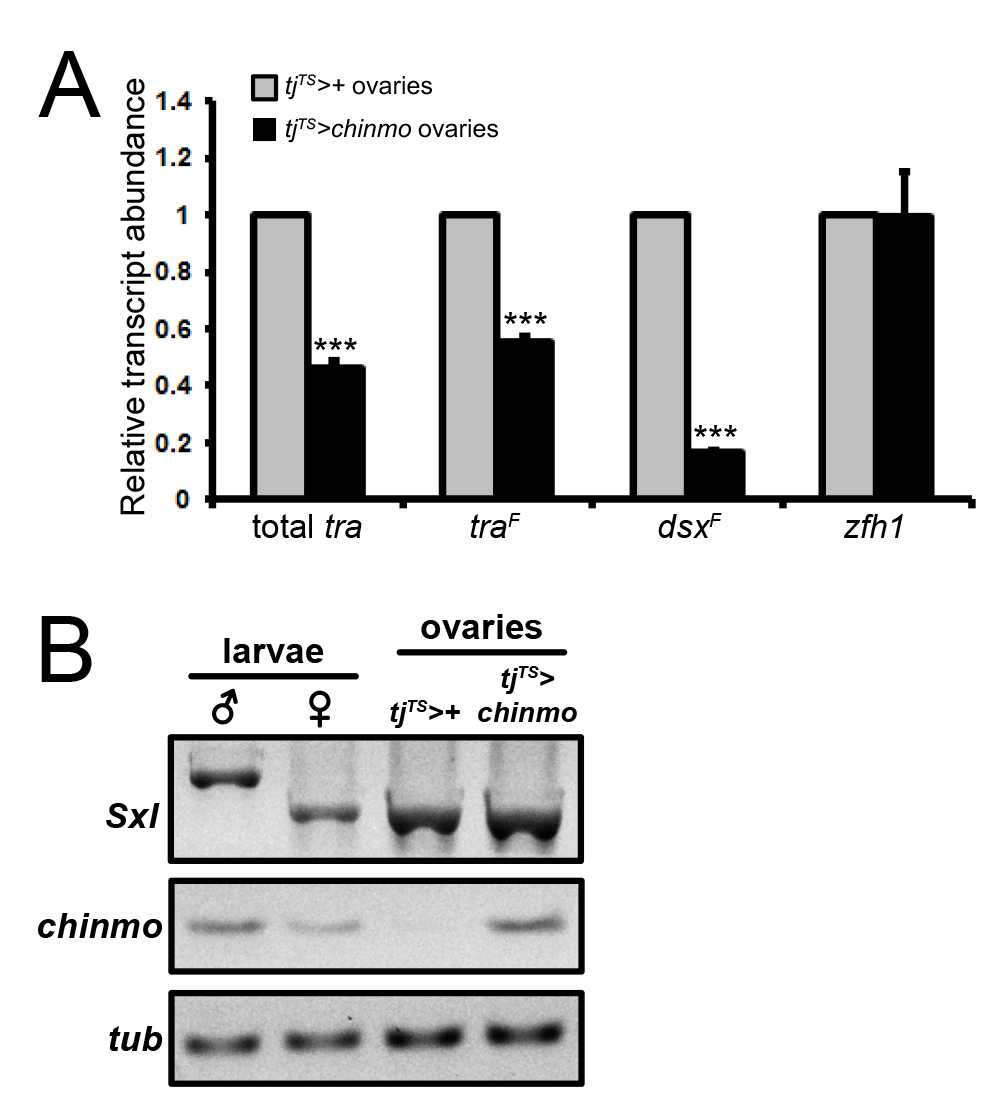

Supplement: S6 Fig — (A) qRT-PCR analysis of homogenized ovaries demonstrates that mis-expression of chinmo in follicle cells leads to decreased levels of total tra, traF, and dsxF. Lower transcript levels were not due to a change in the relative abundance of somatic cells, as zfh1 levels were unaffected in tjTS>chinmo ovaries. The values were normalized to tubulin. Data are presented as the mean of three biological replicates. *** denotes p<0.001 as determined by two-tailed Student’s t-test. Error bars represent SEM. (B) Semi-quantitative RT-PCR on RNA extracts from 5 male or 5 female larvae (first two lanes), tjTS>+ adult ovaries (third lane), and tjTS>chinmo adult ovaries (last lane). RNA from male larvae express SxlM (first lane), while RNA from female larvae express SxlF (second lane). Both tjTS>+ (third lane) and tjTS>chinmo (last lane) ovaries express SxlF exclusively. SxlEM primers were used to differentiate between SxlM and SxlF mRNA isoforms in this experiment. α-tubulin (tub) was used as a loading control. (TIF) [file pgen.1007203.s007.tif]

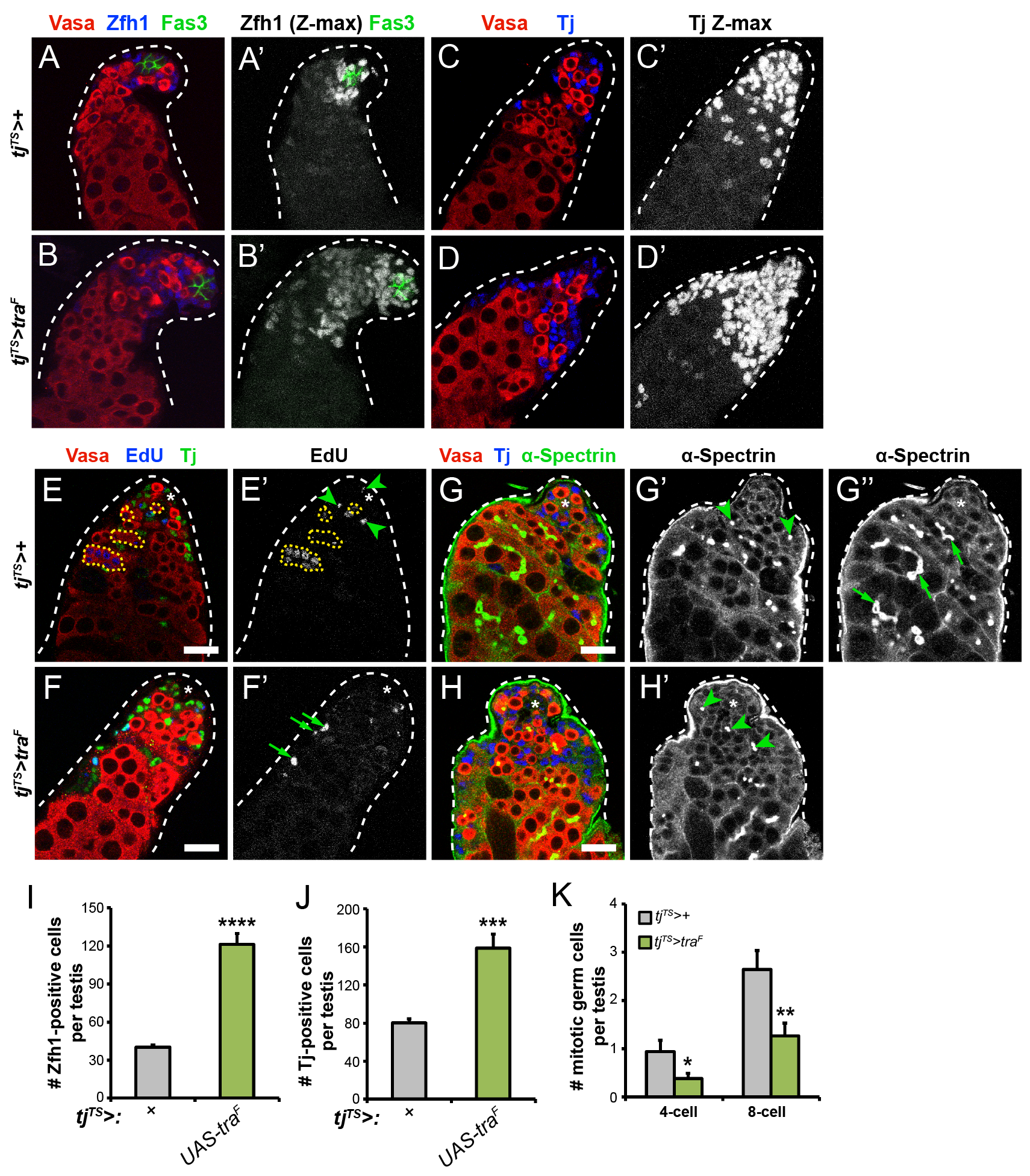

Supplement: S7 Fig — (A-B) Zfh1 (blue) expression in tjTS>+ (A) versus tjTS>traF (B) testes. A and B represent single Z slices; A’ and B’ show maximal Z-projections (Z-max) of Zfh1-expressing cells in the entire confocal stack. Fas3 (green) marks the niche. (C-D) Tj (blue) expression in tjTS>+ (C) versus tjTS>traF (D) testes. C and D represent single Z slices; C’ and D’ show Z-max projections of Tj-expressing cells. (E-F) EdU (blue)-labeled tjTS>+ (E) and tjTS>traF (F) testes. EdU-positive spermatogonial cysts are outlined. Tj (green) marks cyst cells. Arrowheads (E’) point to EdU-positive CySCs. Arrows (F’) point to EdU-positive differentiating cyst cells away from the niche. Asterisk marks the niche. (G-H) Visualization of germ cell stages in tjTS>+ (G) and tjTS>traF (H) testes. α-spectrin (green) marks fusomes, which are dot- and dumbbell-shaped in early germ cells (G’, arrowheads) and become branched in later differentiating spermatogonia (G”, arrows). Note that the niche is not in the plane in G’. Tj (blue) marks cyst cells. Arrowheads in H’ indicate spermatogonia away from the niche that have dot and dumbbell shape fusomes in tjTS>traF testes. Asterisk marks the niche. (I-J) Quantification of Zfh1-expressing (I) and Tj-expressing (J) cells in tjTS>+ (gray bars) versus tjTS>traF (green bars) testes. tjTS>traF testes contain significantly more Zfh1-expressing and Tj-expressing somatic cells than tjTS>+ testes, as determined by single-factor ANOVA. (K) Quantification of EdU-positive germ cells upon somatic traF mis-expression. tjTS>traF testes contain significantly fewer EdU-positive 4-cell and 8-cell spermatogonia than tjTS>+ testes. For quantifications, * denotes p<0.05; ** denotes p<0.01; *** denotes p<0.001; **** denotes p<0.0001 as determined by single-factor ANOVA. Quantification data are presented as mean ± SEM. Vasa (red) marks the germline in A-H. Scale bars = 20 μm. (TIF) [file pgen.1007203.s008.tif]

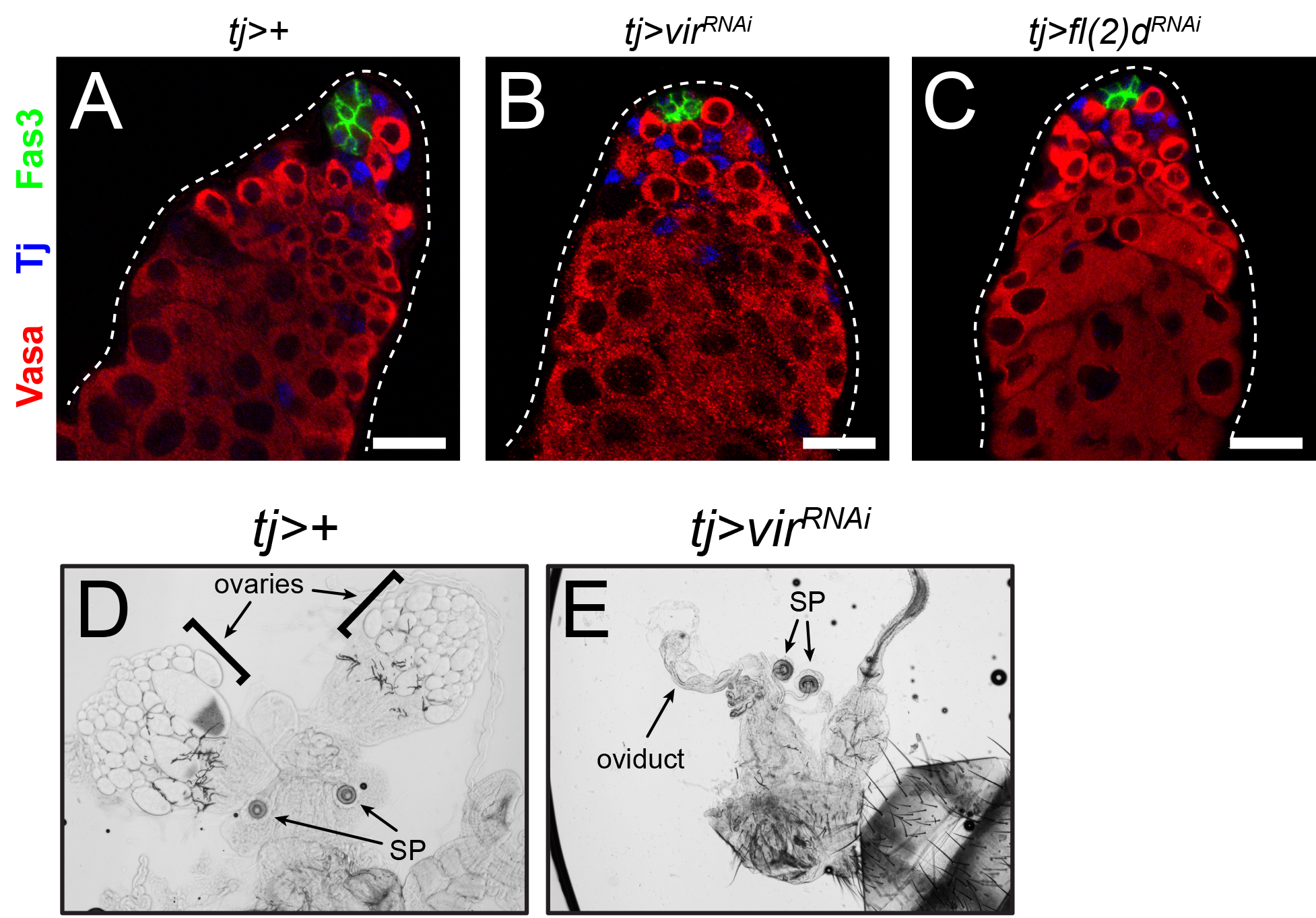

Supplement: S8 Fig — (A-C) tj>virRNAi (B) and tj>fl(2)dRNAi (C) testes resemble control tj>+ (A) testes, showing no overt defects in testis development or spermatogenesis. Vasa (red) marks the germline, Tj (blue) marks somatic cells, and Fas3 (green) marks niche cells. Scale bars = 20 μm. (D-E) Reproductive structures in adult tj>+ (D) and tj>virRNAi (E) females. Ovaries (D, brackets) and accessory structures like spermathecae (SP) (D, arrows) can be observed in tj>+ females. Ovaries, but not somatic accessory structures like SP and oviduct, fail to develop in females lacking vir in the somatic gonad (E, arrows). (TIF) [file pgen.1007203.s009.tif]
